# Secondary Somatosensory Cortex Is Required for Learning but Not Execution of a Tactile Discrimination

**DOI:** 10.1111/ejn.70390

**Published:** 2026-01-29

**Authors:** Anurag Pandey, Sungmin Kang, Nicole Pacchiarini, Hanna Wyszynska, Zena Masseri, Joseph O'Neill, Robert C. Honey, Kevin Fox

**Affiliations:** ^1^ School of Biosciences Cardiff University Cardiff UK; ^2^ School of Psychology Cardiff University Cardiff UK

**Keywords:** behavioural learning, behavioural plasticity, cortical plasticity, network activity, whisker/barrel cortex

## Abstract

The relationship between primary (S1) and secondary (S2) somatosensory cortex is not well understood, and the role of S2 in somatosensory function is not well defined. To test the role of S2 and its interplay with S1 in learning a texture discrimination, we reversibly inhibited primary (S1) and/or secondary somatosensory cortex (S2) bilaterally using DREADDs and measured the effect on the ability of mice to learn a whisker‐dependent tactile discrimination. Freely moving mice foraged in an arena that contained two bowls, one of which contained a buried food reward. The bowls could only be distinguished by the texture on the outer surface. DREADD‐mediated inhibition suppressed sensory responses and disrupted network activity in the cortical area in which DREADDs were expressed. We found that both S1 and S2 were critical for learning the tactile discrimination. Tactile learning in naive mice required normal S2 function during the learning phase but not during the post‐training consolidation phase of approximately 6 h. Furthermore, S2 was only required during learning. Once expert levels of discrimination had been attained, S2 was not required for execution of the learned discrimination. The role of S2 was confined to tactile learning and was not required for olfactory discrimination. Our findings suggest that S1 and S2 interact when learning a new tactile discrimination, but the learned skill eventually becomes independent of S2.

## Graphical abstract

The relationship between primary somatosensory cortex (S1) and secondary somatosensory cortex (S2) during learning is not clear. Here, we show that when confronted with a texture discrimination using the whiskers, S2 is required during learning but not after learning. Left: The mouse learns that it can find a hidden food reward in one of two pots that are only distinguishable by their surface texture in the dark. Right: Learning is rapid, taking 72 trials spaced over 2–3 days (left hand graph, blue line), but only if S2 is normally active. Inhibition of S2 using DREADDs prevents learning in a naive mouse (red line). However, once the discrimination is learned, inactivating S2 has no effect on performance (right hand graph).

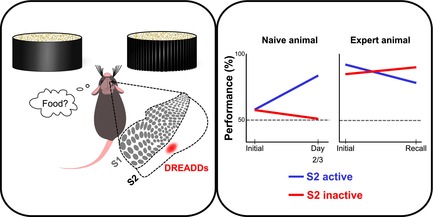

## 1 Introduction

The neocortex contains two main representations of the body surface, one in primary somatosensory cortex (S1) and one in secondary somatosensory cortex (S2). The two areas are closely linked by feedforward and feedback connections and both receive input from the somatosensory thalamus (Aronoff et al. 2010; Minamisawa et al. 2018). It is not clear what the advantage of the arrangement might be. It has been suggested that the two areas may process lemniscal and paralemniscal information in parallel (Kwegyir‐Afful and Keller 2004). Conversely, it is possible that S2 elaborates or integrates tactile features conveyed by S1.

Sequential processing by cortical areas can increase the complexity of receptive fields and simultaneously increase the invariance of what is represented to the point where objects can be coded and therefore identified independent of context (Riesenhuber and Poggio 1999). Information compression is also useful in preprocessing stimuli and any corresponding associations for memory storage. It is possible that S2 both preprocesses information for memory and acts as a link between S1 and higher order cortical areas leading eventually to the hippocampus.

Studies in primates have suggested that tactile memory might be subserved by a multisynaptic pathway from primary somatosensory cortex (S1) to secondary somatosensory cortex (S2) to ‘ventromedial limbic areas’ (Mishkin 1979). This pathway has been termed the ‘ventral stream’ for the somatosensory system to distinguish it from the ‘dorsal stream’ for action, which runs from S1 to motor cortex to frontal cortex (Kaas et al. 2011). There are already indications that a ventral stream from the somatosensory system to the limbic system might exist in rodents. Information from the whiskers finds its way to the hippocampus (Itskov et al. 2011) and anatomical pathways do exist linking S1 to S2 to perirhinal cortex and hence to entorhinal cortex and hippocampus (Burwell and Amaral 1998; Agster and Burwell 2009). However, so far there are few studies aimed at testing the hypothesized pathway's function. The advent of reversible inhibitory methodology and genetic manipulation afford the opportunity to examine the proposed pathway for tactile memory in the mouse and to do so more accurately than with earlier cortical ablation methods.

To test whether a ventral stream for tactile information processing and tactile learning might exist in rodents, we measured the effect of functionally lesioning S1 or S2 on the ability of mice to learn a tactile texture discrimination. We used a behavioural paradigm that required two textures to be discriminated in the dark by freely moving animals using their whiskers (Pacchiarini et al. 2020). Rather than physically ablate cortical areas, we used excitatory DREADDs expressed in inhibitory neurons to produce a time‐limited inhibition of either S1 or S2 during or just after the training period. Our studies show that both S1 barrel field and the whisker area of S2 are essential for texture discrimination learning in mice. Our findings show that S2 is crucial for the initial stages of learning and may be involved in initial recall once the discrimination is learned.

## 2 Materials and Methods

### 2.1 Subjects

We studied C57Bl/6Jax (Charles River, UK) mice and homozygous PV‐Cre strain mice (University of Bristol, UK) of adult age (> 3 months). The number of mice in each experiment was as follows: Experiment 1 (*n* = 24), Experiment 2 (*n* = 38) and Experiments 3 and 4 (involving the same mice, *n* = 18). In addition, the time‐course and effect of DREADD mediated inhibition was studied in five mice. Mice were housed individually and placed on food restriction 1 week before starting behavioural training to bring their weight to 87%–90% of their starting weights. All procedures followed the Animals (Scientific Procedures) Act 1986 and were conducted according to ARRIVE guidelines.

### 2.2 Group Treatments and Rationale

In Experiment 1, to control for the effects of transgenic expression in PV cells and any general effects of CNO, we used an experimental design involving either DREADD or GFP expression and either CNO or saline administration. Thus, 30 min before texture discrimination training sessions, mice received an i.p. injection of either CNO hydrochloride (HelloBio, UK) dissolved in saline (3.5 mg/kg of bodyweight) or saline alone. One group with DREADD injections received CNO (group Tx‐Dd‐CNO; *n* = 8) and the other received saline (group Tx‐Dd‐Sal; *n* = 4). In mice expressing GFP, one group received CNO (group Tx‐GFP‐CNO; *n* = 4) and one received saline (group Tx‐Dd‐Sal; *n* = 4). To assess any general effects of the combination of DREADDs and CNO (e.g., on motivation or behaviour), a further group of mice with DREADDs received CNO 30 min before odour discrimination training (group Od‐Dd‐CNO; *n* = 4), which has many of the same general features as the texture discrimination training, but does not require texture processing.

In Experiment 2, 30 min before either texture or odour discrimination training, groups of mice with DREADDs in S2 received either CNO or saline injections: groups Tx‐Dd‐CNO (*n* = 10), Tx‐Dd‐Sal (*n* = 7), Od‐Dd‐CNO (*n* = 5) and Od‐Dd‐Sal (*n* = 4). GFP groups were not included in Experiment 2 given the results from Experiment 1 (see below). Some mice received DREADDs in S2 that encroached on S1 and either received CNO (8) or saline (2) and these were analysed as a separate case.

In Experiment 3, where the role of S2 in memory consolidation and recall was tested, two groups of animals were used. In the first group, S2 was injected with DREADDs (*n* = 10), while the control group did not receive DREADD injection in S2 (*n* = 8); both the groups were treated similarly with CNO.

In Experiment 4, the same mice were studied further; once they had reached expert levels of performance over 2 days of training, CNO was injected 30 min before testing on the third day.

### 2.3 Surgery

Virus injection surgeries were performed on 6‐ to 10‐week‐old mice using coordinates based on a standard mouse brain atlas (Paxinos and Franklin 2008) as described previously (Fox et al. 2018). S1 or S2 was silenced bilaterally by excitatory DREADDs expressed in the inhibitory neurons. DREADDs were delivered by either pAAV‐hDlx‐GqDREADD‐dTomato‐Fishell‐4 (Addgene, 83897‐AAV9) virus (DREADD‐Fishell) in wild‐types, or pAAV‐hSyn‐DIO‐hM3D(Gq)‐mCherry (Addgene 44361‐AAV9) in PV‐Cre mice. Following DREADD activation by CNO, inhibitory neurons increase their firing rate thereby decreasing the excitatory network activity of the infected area. S1 injections were aimed at the D2, D6 and B2 barrels. The behavioural and electrophysiological reaction to CNO in DREADD expressing areas were similar in PV‐Cre and C57Bl/6‐Jax mice and were thus combined for the purpose of analysis (see Sections 3.1.4 and 3.2.1). The viral loads and injection coordinates used are given in Table 1.

**TABLE 1:** Locations and concentrations for viral injections.

Stereotaxic coordinates			Virus amount	
Posterior from bregma (mm)	Lateral from midline (mm)	Ventral from dura (mm)	Volume (nL)	Concentration (vg/mL)
**Injection of GFP in S1**				
1.3	3.0	0.4	250	AAV‐FLEEX‐GFP (1 × 10^11^)
1.1	3.3	0.4	250	AAV‐FLEEX‐GFP (1 × 10^11^)
1.6	3.2	0.4	250	AAV‐FLEEX‐GFP (1 × 10^11^)
**Injection of DREADDs in S2**				
1.0	4.3	1.3	200	Flexed DREADDs **OR** GqDREADD‐Fishell (1 × 10^11^)
1.2	4.5	1.0	200	Flexed DREADDs **OR** GqDREADD‐Fishell (1 × 10^11^)
1.3	4.2	1.3	100	Flexed DREADDs **OR** GqDREADD‐Fishell (1 × 10^11^)
**Injection of DREADDs in S1**				
1.3	3.0	0.4	250	Flexed DREADDs **OR** GqDREADD‐Fishell (1 × 10^11^)
1.1	3.3	0.4	250	Flexed DREADDs **OR** GqDREADD‐Fishell (1 × 10^11^)
1.6	3.2	0.4	250	Flexed DREADDs **OR** GqDREADD‐Fishell (1 × 10^11^)

*Note:* Top rows: Three injections in each animal of virus expressing GFP into the S1 cortex as a control for the effects of expression (AAV1‐CAG‐Flex‐eGFP‐WPRE‐bGH, Penn Vector Core). Middle Rows: Three injections in each animal of virus expressing DREADDs into S2 to excite parvalbumin‐positive GABA‐ergic neurones in PV‐Cre mice (pAAV‐hSyn‐DIO‐hM3D(Gq)‐mCherry, Addgene) or to excite GABA‐ergic neurones in wild‐types (pAAV‐hDlx‐GqDREADD‐dTomato‐Fishell‐4, Addgene). Bottom rows: Three injections in each animal of virus expressing DREADDs in S1 (viruses as for S2).

### 2.4 Acute In Vivo Electrophysiology

Acute in vivo electrophysiological measurements were performed to quantify the pharmacokinetics of DREADD activation using CNO. Anaesthesia was induced with isoflurane (4% in O_2_) and maintained with urethane (150 mg/mL, Sigma‐Aldrich, USA) and acepromazine maleate (2 mg/mL, Elanco, UK) injected intraperitoneally at a dose of 1.5 mg/g body weight. Urethane was administered by first giving 70% of the dose and then topping up based on the hind limb withdrawal reflex, respiration rate and, once recording, spontaneous cortical activity (Friedberg et al. 1999) in areas unaffected by DREADD. Body temperature was monitored using a rectal thermistor and maintained at 37°C by a heating pad. Mice were secured in a stereotaxic frame (Narishige International, London, UK). For Neuropixels recordings, an oval channel of the cranium was thinned (major axis 2 mm, minor axis 1 mm) and a small opening made just large enough to allow the electrode to be inserted at an angle of 30° from vertical. Electrodes were positioned based on the location of the virus injection sites for each animal and ranged from 1.75–2.25 mm lateral to the midline and 0.7–1.5 mm posterior to bregma. The Neuropixels 1.0 probe (IMEC, Belgium) was inserted slowly at an angle to the depth of 3–3.2 mm over a period of 1–1.5 h, with the narrow edge of the probe facing laterally and the electrode‐bearing side facing rostrally. We used the factory configuration for the probe and recorded using Bank A. Data were acquired using OpenEphys (v0.5), with the probe connected to a computer via the acquisition chassis of a National Instruments board (National Instruments, USA).

Whisker stimuli were applied at 0.2 or 1.0 Hz after probe insertion using a piezo‐electric stimulator (Physik Instrumente, UK) (Fox et al. 2018). For Neuropixels recordings in S1 and S2, the stimulated whisker was selected based on the peak evoked local field potential (LFP), unit sensory responses and number of channels recording a sensory response. Responses were sought in S1 and S2 to stimulation of the same whisker. The electrode was painted with DiI prior to insertion in order to visualize the electrode track from post‐mortem histology. Neuropixels probe penetrations spanned across more than one barrel‐column and electrode tracks were reconstructed from DiI staining in post‐mortem tissue (Figures 3 and 5).

### 2.5 Analysis of In Vivo Electrophysiology Data

Individual files covering different periods of recording were concatenated for analysis. Spikes were sorted using Kilosort 2.5 (Pachitariu et al. 2016). After manual curation with Phy2 (Rossant and Harris 2013), spike clusters were allocated to the channels with the largest average peak‐to‐peak amplitude. Units with clear refractory period (±1.5 ms) were saved for further analysis of the resultant time series using in‐house scripts written in Matlab and C.

### 2.6 Cluster Analysis

To cluster cells in layer 4 of S1 into subtypes we calculated the first moment of the autocorrelation, which provides a measure of firing mode, along with two waveform features; spike width and spike asymmetry (Csicsvari et al. 1999; Bartho et al. 2004; Sirota et al. 2008). These data were passed to a hierarchical clustering algorithm (Ward.d2, R) to separate the neurons into groups based on these features (Ward 1963).

The cluster analysis produced two groups of layer 4 neurons, *n* = 48 and *n* = 214. The first group represented 18.3% of the neurons, which tended to include higher firing rates during baseline (3.1 Hz ± 0.8), with regular firing patterns (first moment of the autocorrelation, 26.9 ms ± 0.5), consistent with interneuron firing. The second group (81.6%) had significantly lower rates during baseline (1.2 Hz ± 0.1, *p* < 0.005, Mann–Whitney *U*, *z* = 2.74) and a greater tendency towards firing bursts with shorter ISI (first moment of the autocorrelation, 20.6 ms ± 0.5, *p* < 0.000001, Mann–Whitney *U*, *z* = 5.5), as might be expected from a population of regular and burst firing excitatory cells. We will therefore refer to group 1 as ‘interneurons’ and 2 as ‘pyramidal cells’. Of this pool of cells, 31 interneurons and 70 excitatory cells were recorded in layer 4 of animals injected with ‘Pan‐GABA’ DREADD, while 17 interneurons and 144 excitatory cells were recorded in ‘PV‐Cre’ mice.

### 2.7 Sensory Responses Under Anaesthesia

Not all neurons recorded responded to the particular whisker we chose to stimulate. To identify the subset of ‘responding’ neurons, we constructed the cross‐correlation between stimulation time points and spike trains for each cell (1‐ms bins). Spike counts established in the 200 ms prior to stimulation were used to estimate a baseline rate. Cells with a peak rate in the 3‐ to 50‐ms time‐window after stimulation that exceeded 2 SD of the mean baseline rate were classified as responding. To calculate the impact of DREADD activation on these responding cells, we calculated their firing rates in 3‐ to 50‐ms windows following stimulation, before and after CNO injection. To quantify this change, we used a change in rate score (see equation below), which ranges from −1 to 1, where zero indicates no change, a negative value a decrease and a positive value an increase in firing rate.

$$∆=\frac{\text{Firing rate after}\;\mathrm{CNO}-\text{firing rate before}\;\mathrm{CNO}}{\text{Firing rate after}\;\mathrm{CNO}+\text{firing rate before}\;\mathrm{CNO}}$$

### 2.8 Spontaneous Activity Under Anaesthesia

Spontaneous firing rates were calculated during the 5 s interstimulus interval, excluding the first second post stimulation (i.e., 4 s epochs) to minimize effects of whisker stimulation. Only cells with rates > 0.2 Hz in the period before or after CNO were included. The resultant spontaneous firing rates from these neurons were used to calculate the ∆ rate score, as in the equation above.

To gauge the time course of DREADD action after a single CNO injection, firing rates (spikes/s) were calculated in 5 min overlapping windows (with the first second after each stimulation excluded), commencing at each stimulation. We calculated the mean baseline firing rate for each cell during the 25 min prior to CNO injection and transformed the firing rates in each window into a score (window rate—mean)/sum (Figure 3E). Similarly, the return to baseline was calculated using a window in the final period of recording. The absolute value of this score denotes the magnitude of the change in instantaneous rate from baseline.

The effect of DREADD on Gamma power (30–80 Hz) was calculated during the inter‐stimulus interval (excluding the first second after stimulation). For a given stimulation, a 3.26‐s window was divided into four nonoverlapping windows (819.2 ms, hamming), in which gamma power was estimated and averaged. We evaluated the last 300 stimulations prior to CNO, and the last 300 windows recorded after CNO injection. We then selected eight channels from the most superficial portion of S1 and a further eight from the deepest portion of S2 in a recording. These channels were evenly distributed across 400um of tissue. The mean and SD of gamma power prior to CNO was established, and the post‐CNO gamma power expressed as a *z*, for each stimulation, on each these electrodes. Delta power (0.5–4 Hz) was calculated in 13.1 s overlapping windows, with a 50% overlap in the 60 min prior to, and after, CNO injection. Like the gamma power analysis, the mean and SD of delta power in the period before CNO (here 60 min) was used to transform the power of the signal into a *z* score. Thus, *z* scores from periods after CNO represent an instantaneous change in power from base line. Averages of these instantaneous scores provided mean change in power, for each electrode (the same electrodes selected for gamma analysis).

### 2.9 Analysis of Changes in Firing Rate Distributions

Changes in firing rate (∆rate, see equation above) were calculated between the first and second half of the period before CNO administration (control period) for all cells. These rate changes yielded a distribution centred on zero with a standard deviation of 0.07–0.17 (S1, spontaneous activity, three mice; Figure 3E), 0.16–0.23 (S1, evoked activity, three mice; Figure 3H), 0.05–0.1 (S2, spontaneous activity, two mice; Figure 5D) and 0.16 (S2 evoked activity, two mice; Figure 5E). Following CNO administration, we defined neurons decreasing their firing rate as those 2 SD below the control distribution mean for that animal and those increasing their firing rate as those neurons 2 SD above the control distribution mean for that mouse. For descriptive statistics, we calculated average increases and decreases in firing rate for the two subpopulations. To judge statistical significance between control and CNO periods, we applied the Kolmogorov–Smirnov (KS) test (Prism, GraphPad Software, Boston MA) to the whole distribution for each animal.

### 2.10 Analysis of Burst Firing and Waveform Properties

To establish whether neurons inhibited (likely excitatory cells) or excited (likely interneurons) by DREADD activation represented separate populations, we evaluated the spike train and waveform characteristics of each cell. Bursting, a characteristic of some excitatory cell populations, was quantified by first calculating each neurons spike autocorrelation [±30 ms, bins = 0.5 ms] and establishing the first moment of the resultant histogram (Csicsvari et al. 1999). Conversely, interneurons tend to have asymmetric waveforms (Bartho et al. 2004). Spike asymmetry was measured by the difference in the peak amplitude before and after the spike trough, divided by the sum (Sirota et al. 2008).

### 2.11 Histology

Upon completion of the experiments, mice were given a lethal dose of pentobarbital (Euthatal, Boehringer Ingelheim) and perfused through the heart to exsanguinate and then fixate the brain. For tangential sections, brains were blocked to remove frontal and occipital cortex. For horizontal sections, subcortical structures were carefully removed from the fixed brain and the cortex gently flattened between two microscope slides separated by modelling clay or blue tack before preserving overnight in paraformaldehyde (4%)–sucrose (20%) solution and thereafter in PBS–sucrose (20%) solution. Horizontal sections (35 μm) of the cortex were cut and the sections destined for immuno‐histochemistry were stored at −20°C in a cryoprotectant solution (50% sucrose, 1% polyvinyl pyrrolidone and 30% ethylene glycol in 0.1‐M PBS). The location of DREADD expression was determined using the native Tdtomato/mCherry signal from the DREADD virus and staining the thalamic axon with Cytochrome oxidase or VGlut2 antibodies to visualize the barrel fields. DREADD injections were located by overlaying the images of stained sections on a standard flattened cortical map (Figures 4, 6 and 7).

#### 2.11.1 cFos Expression Assay

Mice undergoing behavioural training and/or treatment with CNO were left in a dark room in their home cages for 90 min to enhance the cFos protein expression prior to anaesthesia as described above. For immuno‐histochemistry, individual floating sections were thoroughly rinsed in PBS solution and blocked for 1 h with 2% goat serum and permeabilized with 0.05% Triton X‐100 in PBS (PBST). The slices were then incubated at 4°C for 2 days with a mixture of the following antibodies: rabbit anti‐cFos polyclonal primary antibody (1:5000; Synaptic Systems), guinea‐pig polyclonal anti‐VGluT2 primary antibody (1:2000 Synaptic Systems). To confirm the cell type infected with DREADD, a second subset of DREADD injected slices were also incubated in rabbit anti‐PV polyclonal antibody (1:2000; Swant Inc.) and incubated at 4°C for 24 h. After incubation, slices were washed thoroughly in PBST and incubated in a solution of Alexa Fluor 647‐conjugated anti‐rabbit (1:1000; Abcam) and either Alexa Fluor 488 conjugated anti–guinea pig antibody (1:500; Abcam) or Alexa Fluor 568‐conjugated anti‐guinea pig antibody (1:500; Abcam) in the 2% blocking solution for 2 h at room temperature. The second batch of anti‐PV slices were incubated in a solution of Alexa Fluor 488‐conjugated anti‐rabbit antibody. All slices were then washed in PBST and incubated in DAPI (1:15,000; Sigma Aldrich) in PBS for 10 min. Slides were washed in PBS, air dried and mounted in Fluoromount aqueous mounting medium (Sigma Aldrich) and cover‐slipped using Vectashield (Vector Laboratories). The following control solutions were used for both protocols: (1) a solution without the primary antibody, (2) a solution without the secondary antibody and (3) a solution without any antibody. No cellular fluorescence was detected with these control solutions (data not shown).

### 2.12 Behavioural Training

**FIGURE 1:**
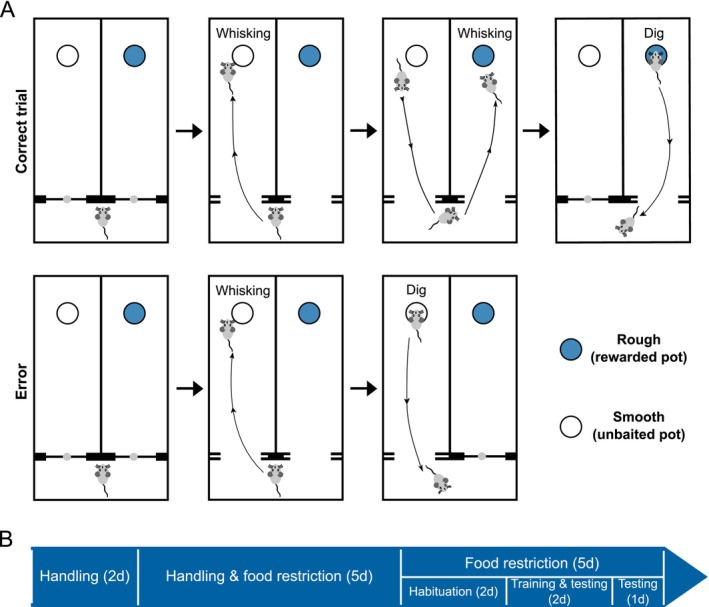
(A) The arena used for discrimination training and examples of a mouse making a correct choice (upper sequence) and an incorrect choice (lower sequence). Upon leaving the holding compartment, the mouse could move freely between the two bowls in the choice compartments, but access to the holding compartment was removed by closing the guillotine door. For the correct choice: The mouse chooses to dig in the sawdust filled bowl on the right which contains the food reward, a correct choice. When the mouse initiates digging, the door to the neighbouring compartment is closed so that the mouse cannot dig in both bowls. At this point, the door to the holding area is opened so that the mouse can return; this area also contains a water bowl (not shown). For an incorrect choice: The mouse digs in the unrewarded bowl, whereupon access to the neighbouring compartment is prevented and access to the holding area remains available. For the texture discrimination, the outer surfaces of the bowl (e.g., grooved but not smooth) indicated the presence of reward; whereas for the odour discrimination, the odour of the sawdust (e.g., cinnamon but not ginger) indicated the presence of reward. (B) An infographic illustrating the timeline for different stages of Experiments 1 and 2.

The apparatus used for behavioural training and features of the training procedure are depicted in Figure 1 and described in detail in (Pacchiarini et al. 2020), see also Movie S1. Briefly, the arena was placed on a table in a small experimental room illuminated with dim red light. The arena consisted of a waiting compartment (20 cm × 10 cm), allowing access to the two choice compartments via guillotine doors (15 cm × 10 cm). Cylindrical digging bowls (45 mm in diameter, 25 mm in height) were created using 3D printer technology (Ultimaker B.V., The Netherlands) and RS 3D Printer Filament Polylactic Acid (PLA; 2.85 mm 1 kg). A drinking bowl printed using Wood PLA was placed in the holding compartment filled with water. The bowls in the choice compartments could be baited with a small piece of Chocorice (glucose syrup and cocoa powder (5.5%) coated rice flakes (76.5%), Crownfield, UK). This reward was concealed in sawdust (mixed with 2% Chocorice blended to form a dust to mask the scent associated with reward), which meant that mice were required to dig in order to retrieve the hidden reward. During texture discrimination training, the outer surfaces of the bowls were discriminated by their texture (grooved or smooth); and during odour discrimination training, the bowls had the same texture (smooth) and were discriminated by the odour of the sawdust (the presence of 0.5% ginger or 0.5% cinnamon; cf. Davies et al. 2013; Grieves et al. 2016).

#### 2.12.1 Pretraining

During the first 2 days, mice were handled by the experimenter in their home cage. Over the subsequent 5 days, they continued to be handled and gradually food deprived to 87%–90% of their initial weights, which was then maintained throughout behavioural training. In the final two of these 5 days, a baited sawdust‐filled bowl was placed in the home cage to familiarize the mice with the bowl and digging process. On each of the next 2 days, mice received a single session in which they were placed in the arena. During the first trial, they were allowed to explore the empty arena for 10 min. In the next two trials, they were placed in the arena to explore the identically textured rewarded bowls placed in both the chambers. This texture continued to be rewarded during discrimination training.

#### 2.12.2 Discrimination Training

Mice received discrimination training during the next 3 days. On the first 2 days, mice received 4 ‘warm‐up’ trials in which they explored both the choice chambers, with the rewarded bowl in one chamber and the unrewarded bowl in the other. Each trial had a minimum duration of 5 min and continued until the mice had dug in both the bowls. On the next 24 trials of the first 2 days of training, once the mouse initiated digging it was isolated in the choice compartment containing that bowl by closing the door to the other compartment. These trials lasted for at least 1 min or until the mouse dug in one of the bowls. Trials where mice did not dig in any bowl within 5 min were aborted and excluded from further analysis. On the final day of training, mice received a further 24 trials without the initial ‘warm‐up’ trials. The position of the rewarded bowl varied pseudo‐randomly between the trials and was evenly counterbalanced for each mouse. The analysis of discrimination accuracy was pooled over the trials where mice were only allowed to dig in one bowl.

#### 2.12.3 Consolidation Experiments

The role of S2 in the consolidation of texture discrimination learning was tested by silencing S2 after the mice were trained for texture discrimination as described earlier. During the first 3 days of these experiments, CNO was injected immediately after texture discrimination training in two groups of mice, one with DREADD injection in S2 and other without DREADD injected in S2. On the fourth day these mice were tested for texture discrimination under reversed reward contingencies and CNO was injected immediately after this ‘reversal testing’. Next day mice were again tested for texture discrimination ability under the same reward contingency as the previous day, but CNO was not injected after the training. After this, the mice were moved to their home cages still under food restriction for 2 days without any behavioural testing.

#### 2.12.4 Expert Level Training and Testing for Task Execution

Two days after the first phase of behaviour experiments, the mice were trained for an extended number of sessions to achieve expert level performance (without any injection of CNO). In these sessions, mice were trained for 10 blocks of four trials instead of the usual six blocks of four trials each in the first phase. This training was repeated for a second day. On the third day, each mouse received one dose of CNO 30 min before the task to test whether silencing S2 had any effect on the ability to recall or perform the task. After behavioural testing, the mice were taken off food restriction and moved to their home cage.

### 2.13 Experimental Design and Statistical Analysis

We made statistical comparisons between cohorts of mice in all four experiments. In addition, we made within‐animal comparisons in Experiments 3 and 4. Details of the statistical analysis are included as they appear. Briefly, parametric statistics were applied for the normally distributed data otherwise nonparametric statistics were used. For parametric tests, ANOVAs were run to test for effects and interactions before using post hoc *t*‐tests. For behavioural data, we used a binomial test to gauge if individual animals learned, while ANOVA were generally applied to ascertain whether a group of animals learned under different conditions. Where ANOVA results are quoted, we use the convention *F*_(number of parameters number of degrees of freedom)_ = *F* value, *p* = value. Note that this results in 1 parameter and 1 degree of freedom for the effect tests as reported using JMP16 statistics software. Paired *t*‐tests were used for within‐animal comparisons. Statistical significance was indicated by *p* < 0.05 and lack of significance for *p* ≥ 0.05 (actual *p* values are reported in the text and figures except when *p* < 0.0001). We used JMP 16 (SAS Software, USA), Microsoft excel, Matlab, GraphPad Prism and Sigma Plot software for data analysis and plotting graphs.

## 3 Results

### 3.1 Experiment 1

**FIGURE 2:**
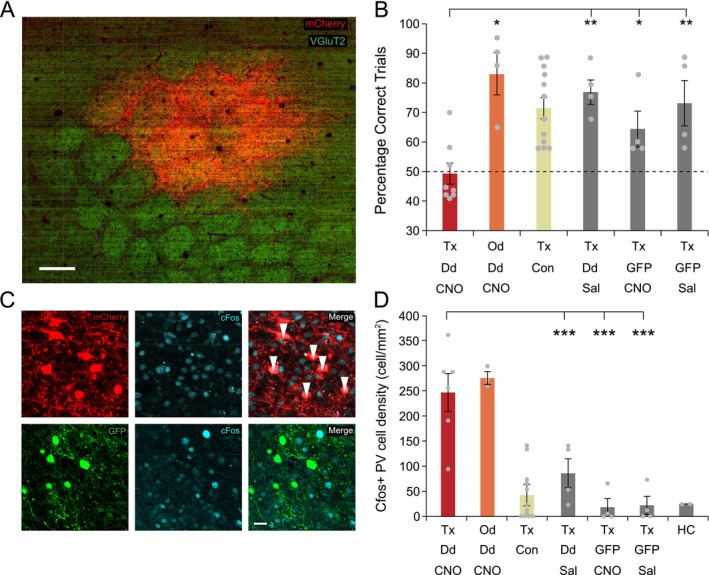
Experiment 1: Effect of inhibiting S1 on texture and odour discrimination learning. (A) Example of DREADD expression in PV cells revealed by co‐expression of mCherry. (B) Mean percentages of correct trials (±SEM) across different conditions (Tx = texture discrimination and Od = odour discrimination; Dd = DREADD and GFP = GFP, CNO = CNO and Sal = saline); with the group designations reflecting these abbreviations. For example, mice in group Tx‐Dd‐CNO perform texture discrimination sessions 30 min after receiving CNO activation of DREADDs. Con = the overall mean (yellow) from the three control groups (shown in grey). (*p < 0.05, **p < 0.005, see Results for statistics). (C) Examples of cFos levels in PV cells containing hM3Dq (top row) versus GFP (bottom row). Note that DREADD expressing PV cells (red) show high levels of cFos expression (top, merge, white arrows) compared with GFP expressing PV cells (bottom merge). Scale bar 15 μm in all panels. (D) cFos positive cell density for the same conditions as shown in B plus one extra condition HC, (home caged) where mice had no interaction with the test arena. cFos density is significantly higher only when CNO administration is combined with DREADD expression (***p < 0.002).

We adapted a rodent discrimination assay (Birrell and Brown 2000) to test tactile texture discrimination learning in mice (Figure 1, Movie S1). To test whether texture discrimination depends on neuronal activity in S1, we activated an excitatory DREADD (hM3D(Gq)) expressed bilaterally in parvalbumin positive interneurons (PV cells) in barrel cortex (Figure 2A) using CNO. The location of DREADD expression was assessed from post‐mortem histology using the native mCherry signal from the DREADD virus and VGlut2 antibodies to stain thalamic axons in the barrel field (Figure 2). Expression of DREADDs in PV cells covered an average area of 500 μm^2^ in each hemisphere and was similar in all treatment groups (*F*_(4,41)_ = 2.32, *p* = 0.075). Approximately 30 min before discrimination training began, we administered CNO i.p. at a dose of 3.5 mg/kg (Pandey et al. 2022). To control for the effects of transgenic expression in PV cells and any general effects of CNO, we used an experimental design involving DREADD or GFP expression and CNO or saline injection.

#### 3.1.1 Texture Discrimination

The mean percentage of correct choices pooled across the three texture discrimination training sessions in Experiment 1 is shown in Figure 2B. It is clear that while the scores for the DREADD injected group receiving CNO (Tx‐Dd‐CNO, red bar) did not differ from chance (i.e., 50%), the scores for the remaining control groups given the same texture discrimination assay (the grey bars) were higher than chance. An ANOVA confirmed that there was a main effect of treatment (*F*_(3,19)_ = 6.904, *p* = 0.003, *η* ^2^ = 0.564), and least significant difference testing showed that the scores for each of the three control groups differed from those in the Tx‐Dd‐CNO group (DREADD plus saline (Tx‐Dd‐Sal) *p* = 0.046, GFP plus saline (Tx‐GFP‐Sal) *p* = 0.001 and GFP plus CNO (Tx‐GFP‐CNO) *p* = 0.004). There were no significant differences between the three control groups, for which the yellow bar represents the mean. Furthermore, while the scores for the mice in the control groups differed significantly from 50% correct, (*t*_(11)_ = 6.014, *p* = 0.001, *d* = 1.736), those from the Tx‐Dd‐CNO group did not, (*t*_(7)_ = −0.189, *p* = 0.856, *d* = −0.067).

#### 3.1.2 Odour Discrimination

In order to test whether inhibition of S1 caused a nonspecific effect on learning and/or other behaviours related to performing the digging task, we tested the effect of DREADD activation in S1 barrel cortex on an odour discrimination (see Methods). The scores for the mice given an odour discrimination are shown in Figure 2B (group Od‐Dd‐CNO, orange bar) and differed significantly from 50% correct (*t*_(3)_ = 4.619, *p* = 0.019, *d* = 2.309) showing clearly that the mice learned the odour discrimination well in the absence of normal S1 neuronal activity.

#### 3.1.3 cFos Expression

To test the effect of CNO administration on DREADDs expressed in inhibitory cells we used cFos immuno‐staining. Analysis was restricted to layer 4 so that the barrel cortex could be identified easily and because we reasoned that inhibition of layer 4 should have a large effect on sensory processing throughout the cortical columns. We found that PV cells significantly increased their cFos expression as a result of CNO‐mediated DREADD activation (Figure 2C,D). Counts of PV cells expressing cFos above a standard threshold level (see Methods) increased 10‐fold following CNO activation both for mice undergoing texture discrimination and odour discrimination mice (red and orange bars, respectively; Figure 2D). A two‐way ANOVA for DREADD (DREADD vs. GFP) and CNO (CNO vs. Saline) revealed an interaction between DREADD and CNO (*F*_(1,1)_ = 5.86, *p* = 0.0296). Post hoc *t*‐tests showed this was due to the DREADD expressing mice treated with CNO being different from all other treatment combinations (GFP + saline *p* = 0.0001, GFP + CNO *p* = 0.0001, DREADD + saline *p* = 0.0015). Conversely non‐PV cells decreased their cFos expression levels significantly compared with controls within the same regions of interest in the same sections (reduction to 79% of control levels, *F*_(1,9)_ = 6.89, *p* = 0.0303, one‐way ANOVA). Of the 5962 cFos^+^ cells we counted within layer 4 of the barrel field, 11.05 ± 3.5% expressed DREADD, giving a rough estimate of the number of PV cells present.

#### 3.1.4 Effect of DREADD on Neuronal Activity in S1

**FIGURE 3:**
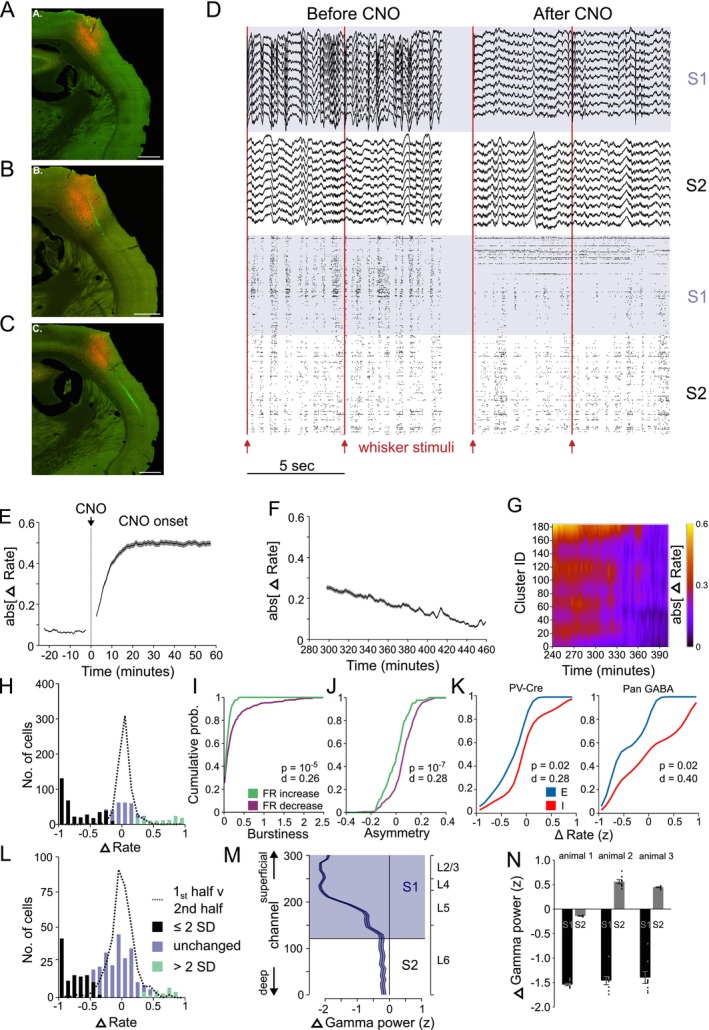
Neuropixels recordings characterizing the effect of activating excitatory DREADD expressed in inhibitory neurons in S1. (A–C) Examples of Gq‐DREADD‐td‐tomato expression in S1; the electrode track is stained in green from the DiI. Scale bar = 500 μm. (D) Local field potential and spike timing of neurons before (left) and after (right) injection of CNO. Top traces (blue background) represent local field potentials taken from S1, lower traces (white background) from S2. Each row of the raster represents an individual neuron, each vertical tick the time of an action potential. Red lines indicate whisker stimulation. (E) The time course of CNO/DREADD on spontaneous firing rate reaches asymptote at ~20 min post CNO injection. Increases and decreases in rate are rectified to plot on the same axis (733 neurons, 3 mice). (F) The change in firing rate returns to baseline after approximately 420 min (517 neurons, 3 mice). (G) Depth profile of changes in firing rate versus time shows most neurons return to baseline after 360 min. The colour code indicates the same Δ‐rate scale shown in Panels E and F (185 neurons, 1 mouse). (H) Distribution of the change in spontaneous S1 firing rate following CNO administration (see Methods). Most cells (50.6%) decreased their firing rate (black 2SD below mean), a few (17.5%) increased (green 2SD above mean) and some were unchanged (blue) (*n* = 733 S1 neurons). Dashed line: change in rate score for the first versus second half of the recording prior to CNO application. (I) The tendency to fire bursts of spikes was greater in the neurons that decreased firing rate (purple) versus those that increased firing rate (green) compared with control (*p* = 10^−5^). (J) Spike waveform asymmetry was significantly higher for neurons decreasing firing rate (purple) versus those increasing firing rate (green) compared with control (blue) (*p* = 10^−7^). (K) The change in firing rate (*z*) of neurons clustered into groups by firing pattern and waveform shape and designated excitatory (E, blue) and inhibitory (I, red) were significantly different in both PV‐Cre mice expressing floxed DREADD (*p* = 0.02, *d* = 0.28, KS test) and wild type mice expressing Pan‐GABA DREADD (*p* = 0.02, *d* = 0.4, KS test). (L) Distribution of the change in evoked S1 firing rate for responses to whisker stimulation. Colours represent the same groups as in H (*n* = 351 neurons). Dashed line: change in rate score for the first versus second half of the recording prior to CNO application. (M) Depth profile of gamma power (30–80 Hz) along the electrode track shows DREADD‐Gq activation in S1 reduced spontaneous gamma power in S1 but not in S2 (see Methods). The mean and SEM are shown for every 8th electrode on the probe spanning the S1 & S2 for mouse 1 (*n* = 37). Reductions in S1 were highly significant (*t*_(299)_ = 4.6, *p* = 0.00037, Bonferoni corrected). (N) Gamma power was strongly decreased during DREADD activation in S1 (black bars) in all three mice tested (*t*_(7)_ = 5.98,1.75,1.15; *p* < 0.0001). S2 gamma power was either unaffected or showed a small increase (grey bars)(*t*_(7)_ = 3.27,1.35,4.65; *p* < 0.0001).

To test the effect of DREADD activation with CNO on neuronal activity, we used electrophysiological recordings with Neuropixels 1 multielectrode probes angled so as to pass through both S1 and S2 (Figure 3A–C). While we saw both synchronous delta‐wave activity and associated bursts of action potentials before CNO injection, both were selectively reduced in S1 and unaffected in S2 following CNO injection (Figure 3D; Figure S1). Simultaneously, in S1, several low firing rate neurons began firing tonically at an average rate of approximately 10–20 Hz.

Neuropixels recordings in S1 showed that these effects occurred within 20 min of CNO administration (Figure 3E) and recovered slowly to baseline values after approximately 420 min (Figure 3F). Note that changes in rate are rectified in this figure so that increases and decreases in rate count in the same positive direction. The penetration profile through S1 showed that most network activity recovered within 360 min (Figure 3G).

Following CNO application, 50.6% neurons showed a reduction in spontaneous firing rate of more than 2 standard deviations from baseline, while 17.5% showed an increase (Figure 3H, 733 neurons, 3 mice). Neurons decreasing their firing rate were characterized by asymmetric spike waveforms and tended to fire in bursts of spikes (Figure 3I,J), both of which are features of excitatory neurons, while neurons increasing their firing rate had symmetric spike waveforms and did not fire in bursts, consistent with them being inhibitory interneurons (Figure 3I,J). Within layer 4 of S1, we analysed 262 neurons using hierarchical clustering (Ward 1963; see Methods) and found 18.3% to be classified as putative inhibitory neurons, which was higher than the 11.05% estimate of PV cells from cFos analysis (Section 3.1.3) and consistent with PV cells comprising a subset of inhibitory neurons.

We found that the same effect was produced by a floxed virus expressing DREADD in PV‐Cre mice as produced by a Pan‐GABA virus expressing DREADDs in WTs. While DREADD activation affected excitatory and inhibitory cells differently *F*_(1,254)_ = 22.10, *p* = 4.24 x 10^−6^, it made no difference which virus type was used (*F*_(1,254)_ = 0.72, *p* = 0.40), and there was no interaction between virus type firing rate (*F*_(1,254)_ = 0.032, *p* = 0.86), indicating that they had a similar effect (whole model ANOVA, *F*_(2,254)_ = 5.4436, *p* = 0.004843). Similarly, analysis of the cumulative distribution functions for the putative inhibitory and excitatory cells identified by clustering algorithms (see Methods) showed the two cell types reacted very differently to DREADD activation (Figure 3K) for both virus types (PV‐Cre *d* = 0.28, df = 159 *p* = 0.02; Pan‐GABA *d* = 0.4, df = 98 *p* = 0.02).

For those cells that decreased their firing rate, the average spontaneous firing rate after DREADD activation was 20.87% of baseline levels. Conversely, those cells that increased their firing rate showed an average 32‐fold increase in firing rate. The large increases were partly due to the low initial firing rates of some of the inhibitory neurons prior to CNO administration (i.e., 0.6 Hz or less). The distribution of firing rates was highly significantly different following DREADD activation for all three mice analysed (*D* > 0.36, *p* < 0.0001, KS test; Figure S3 [top row]).

Sensory responses were affected by DREADD activation in a variety of ways (Figure S4), with cells either decreasing or losing their sensory response all together or, in a small number of cases, increasing their responses (Figure 3L, green). Overall, 32.5% of S1 neurons showed a decrease of more than 2 standard deviations from the baseline period during stimulation. The sensory responses of this group of cells decreased to 14.5% of baseline (Figure 3L, black). A small group comprising 7.1% of neurons increased their sensory responses and on average produced responses 57 fold higher than baseline (Figure 3L, green). The distribution of evoked response levels was highly significantly different following DREADD activation for all three mice analysed (*D* > 0.31, *p* < 0.0001, KS test; Figure 3L, Figure S3 [third row]).

Delta power decreased following activation of DREADD in S1 (Figure S1). Average power fell in all three animals tested (range: 26%–43% of pre CNO oscillatory activity). Delta power did not decrease in S2 in the same animals and instead tended to show increased delta power. Gamma power was also affected by DREADD activation. S1 electrodes showed a profound drop in gamma power in all three mice tested (range: 31%–77.6% of control levels; Figure 3M,N). S2 gamma power was either unaffected or showed a small increase (range 96%–117% of baseline).

### 3.2 Experiment 2

**FIGURE 4:**
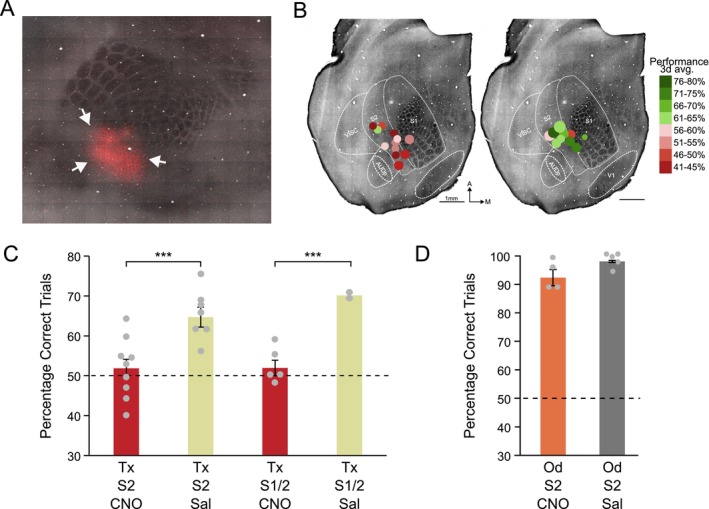
Experiment 2: Effect of inhibition of S2 on texture and odour discrimination learning. (A) Example of Gq‐DREADD‐tdtomato‐Fishell‐4 expression in S2 outside the barrel field in a horizontal section through layer 4. Arrows indicate DREADD expression in S2. The barrel field is stained with cytochrome oxidase. Scale bar = 500 μm. (B) Locations of DREADD expression for individual mice on a standard map of the flattened mouse brain (CNO left, SALINE right). The circles are positioned at the centre of the expression loci with the size indicating the approximate extent of expression and the colours indicating the learning scores for the corresponding texture discrimination assay (see key). (C) Mean percentages of correct trials (±SEM) across different groups of mice with DREADDs expressed in S2 or accidentally in S1 and S2 (S1/2). Mice received injections of CNO or saline (Sal) 30 min before texture discrimination training sessions (Tx) (***p < 0.001). (D) CNO and saline injections 30 min before odour discrimination training sessions (Od) had no effect on learning in groups of mice with DREADDs expressed in S2.

To test whether the secondary somatosensory cortex (S2) is required for learning a texture discrimination, we used the same strategy that we used for barrel cortex (S1) by increasing inhibition in S2 with DREADDs during learning. The location of DREADD expression with respect to S1 and S2 was assessed from flattened cortical sections of each hemisphere (Figure 4A). Post‐mortem histology showed that DREADD expression was confined to S2 in 16 cases and additionally strayed into S1 in seven cases in this cohort (Figure 4B).

#### 3.2.1 Texture Discrimination

We used a Pan‐GABA virus to express DREADD in 9 wild‐type mice and floxed DREADD to express GABA (in PV cells) in 14 PV‐cre mice and found that both viral expression methods had a large effect on learning (*F*_(1,1)_ = 15.11, *p* = 0.001), with no difference between the virus types (*F*_(1,1)_ = 0.06; *p* = 0.79), nor an interaction between the virus‐type and learning (*F*_(1,1)_ = 0.822; *p* = 0.37; whole model *F*_(3,22)_ = 9.58, *p* = 0.0005). Mice were categorized into sub‐groups according to whether DREADD expression was restricted to S2 or also involved S1 (Figure 4B,C). A two‐way ANOVA for DREADD location (S2 only vs. S1 + S2) and treatment (CNO vs. saline) was significant (*F*_(3,23)_ = 9.72, *p* = 0.0001) and gave an effect of treatment on learning (*F*_(1,1)_ = 25.64, *p* = 0.0001) but not location (*F*_(1,1)_ = 0.67, *p* = 0.42). Post hoc *t*‐tests on just the S2 expression cases showed that there was a significant difference between learning in saline controls and CNO treated animals (*t*_(15)_ = 3.81, *p* = 0.0017). Scores for the mice in the DREADD‐CNO group tested for texture discrimination (Figure 4C), did not differ significantly from chance levels of 50% correct (*t*_(9)_ = 0.87, *p* = 0.41; Cohen's *d* = 0.275) while those in the saline group did (*t*_(6)_ = 5.76, *p* = 0.0012; Cohen's *d* = 2.18). Therefore, increased inhibition restricted to S2 significantly affected discrimination learning.

#### 3.2.2 Odour Discrimination

As a control for nonspecific effects on learning and behaviour, we also tested a separate cohort of mice on an odour discrimination. The DREADD expression was confined to S2 in this cohort. We found that increasing inhibition bilaterally in S2 did not prevent the mice learning the odour discrimination. The mice in the DREADD‐CNO group (Figure 4D), differed significantly from chance levels of 50% correct (*t*_(3)_ = 14.79, *p* = 0.0007; Cohen's *d* = 7.40). Mice in the DREADD‐saline group also learned the odour discrimination (Figure 4D) and their scores differed significantly from 50% (*t*_(4)_ = 46.23, *p* = 0001; Cohen's *d* = 20.68) and did not differ from the CNO treated group (*F*_(1,7)_ = 2.11, *p* = 0.197). We therefore conclude that inhibiting S2 does not have a general effect on learning a discrimination.

#### 3.2.3 Effect of DREADD on Neuronal Activity in S2

**FIGURE 5:**
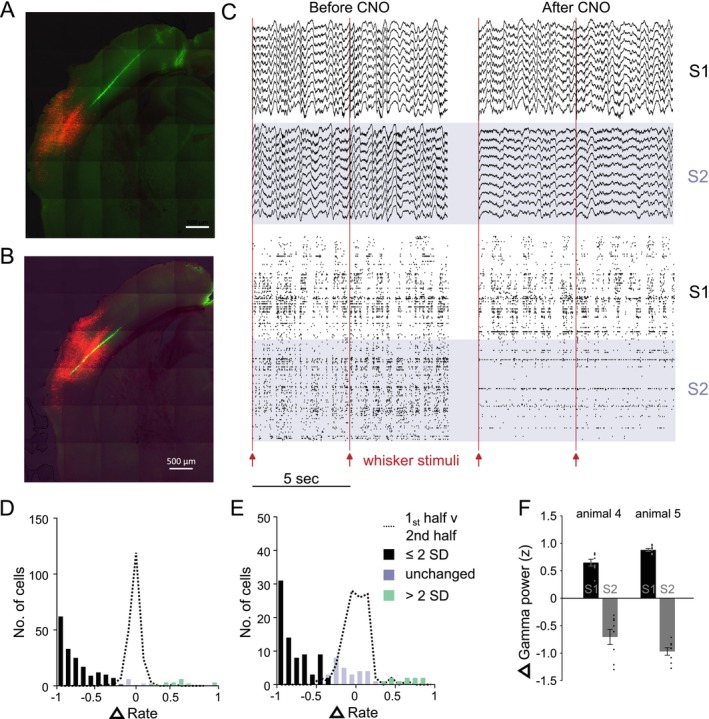
Location of DREADD in S2 and effect on neuronal activity. (A,B) Example of DREADD‐td tomato expression in S2 showing the electrode track in green from DiI on the electrode. (C) Example of the effect on DREADD activation in S2 (blue background) compared with S1 (white background) on local field potentials (top four panels) and action potentials (lower four panels). Note that delta wave activity decreases and network spontaneous activity is disrupted selectively in S2. Red lines indicate whisker stimulation. (D) Distribution of the change in spontaneous S2 firing rate calculated as described in Figure 3 and Methods. (*n* = 206 S2 neurons). After CNO application 85.4% neurons showed a decrease in rate (black bars, < 2SD below the mean), while 9.7% showed an increase in rate (green bars, > 2SD, overlaid in red). The dashed line shows the control period rate change distribution. (E) Distribution of the change in evoked S2 firing rate for responses to whisker stimulation (see Figure 3 and Methods). Most neurons (67.9%) decrease their sensory responses following DREADD activation (black bars) and a few (13.1%) increase their responses (green bars). Dashed line: change in rate score for the first versus second half of the recording prior to CNO application for spontaneous (D) and evoked (E) activity. (F) DREADD‐Gq activation reduces S2 gamma power. Gamma power (30–80 Hz) was measured before and after CNO injection in inter‐stimulus intervals (see Methods and Figure 3L,M). The average gamma power significantly reduced following CNO injection amongst S2 electrodes in both animals (*t*_(7)_ = 7.97 and 15.682, *p* < 0.0005, Bonferroni corrected).

We used electrophysiological recordings to determine the effect of DREADD activation on inhibitory neurons in S2. Recordings were made using Neuropixels 1 multielectrode probes angled so as to pass through both S1 and S2 (Figure 5A,B). Inhibition was even greater for S2 than that achieved in S1 (Figure 3). As with S1, we saw a reduction in burst‐pause spike firing in the delta‐wave frequency range in S2 (Figure 5C) and an increased rate of firing of a small number of neurons. We analysed 206 S2 neurons and found activation of DREADD produced decreases in firing rate in more than 85% of S2 neurons (< 2 standard deviations below baseline firing; black, Figure 5D). A small number of neurons (9.7%) showed an increase in firing rate (> 2 standard deviations above baseline firing; green, Figure 5D).

For those cells that decreased their firing rate, the average spontaneous firing rate after DREADD activation was 15.87% of baseline levels. Conversely, those cells that increased their firing rate showed an average 88‐fold increase in firing rate. As with S1, the large increases were partly due to the low initial firing rates of the inhibitory neurons prior to CNO administration. The distribution of firing rates was highly significantly different following DREADD activation for both mice analysed (*D* > 0.84, *p* < 0.0001, KS test; Figure 5D, Figure S3 [Row 2]).

Sensory responses were also strongly inhibited. Overall, 67.9% of S2 neurons showed a decrease of more than 2 standard deviations from the baseline period during stimulation. The sensory responses of this group of cells decreased to 13.1% of baseline (Figure 5E). A small group comprising 8.9% of neurons increased their sensory responses and on average produced responses 57 fold higher than baseline (Figure 5E). The distribution of evoked response levels was highly significantly different following DREADD activation for both mice analysed (*D* > 0.69, *p* < 0.0001, KS test; Figure 5E, Figure S3).

Delta power decreased in S2 during DREADD activation to approximate 40% of control levels (Figure S1). Gamma power was also severely disrupted by DREADD activation in S2. The *z* scored change in power in S2 ranged between −1.48 and −0.35 (S2; Figure 5F) which represented a drop in gamma power of between 52.5% and 92.2%, when compared with gamma power prior to CNO application. At the same time, S1 gamma power increased, with *z* scored power ranging between 0.36 and 0.94 (S1; Figure 5F), representing an increase of between 109 and 123% of baseline.

### 3.3 Experiment 3

**FIGURE 6:**
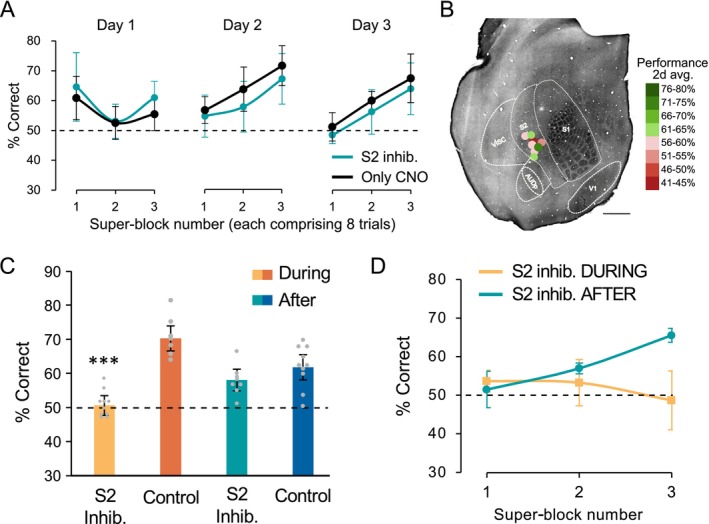
Experiment 3: Effect of Inhibiting S2 after training on learning. (A) Percentage of correct trials per superblock (of eight trials each) for sequential days of training for mice with DREADD located in S2 (turquoise) and controls lacking DREADD (black). CNO is administered to both sets of animals immediately after training on the first and second day. (B) Location of S2 DREADD injections for this group. Discrimination performance is colour coded. Scale bar = 500 μm. (C) Comparison of learning on days 2 and 3 for cases in which S2 is inhibited during learning (light orange bar) or immediately after learning (turquoise bar). Only inhibition of S2 during training affects learning. There is a significant effect of S2 inhibition (*F*_(1,1)_ = 11.706, *p* < 0.0018) and an interaction between S2 inhibition and when inhibition is imposed (*F*_(1,1)_ = 5.422, *p* < 0.0266, two‐way ANOVA). S2 inhibition during learning (yellow) is significantly different from control (orange, CNO but no DREADD) (*F*_(1,16)_ = 18.27, ***p = 0.0007) S2 inhibition after learning (turquoise) is not different from control (blue, *F*_(1,17)_ = 0.554, *p* = 0.467). S2 inhibition during learning (yellow) is the only case not different from chance levels of 50% (*t*_(9)_ = 0.218, *p* = 0.8321). (D) Comparison of the time‐course of learning each day in mice with S2 inhibited after training (but not during the discrimination, turquoise) and those with S2 inhibited during learning (yellow).

Earlier studies suggested that the effect of DREADD inhibition was likely to have extended into the period after the discrimination by several hours (Pandey et al. 2023) and this finding was corroborated by results reported above (Figure 3E,F). As the neuronal activity immediately after training might produce an initial phase of memory consolidation, we wanted to know whether it was required for learning the texture discrimination. Therefore, we trained mice on the texture discrimination for three successive days and administered CNO to activate the DREADD immediately after training was finished each day (Figure 6). Of the 11 mice injected with DREADD in this cohort, post‐mortem histology showed that DREADD expression was confined to S2 bilaterally in 8 cases and these were used in the analysis (Figure 6B).

We found that there were no differences between control animals (that lacked DREADD in S2 but received an injection of CNO) and the S2‐inhibited animals (Figure 6A). Both sets of animals learned the task and showed improvement in performance during the day's set of trials.

A comparison of the effect of inhibiting S2 before the start of training each day compared with inhibition initiated at the end of training each day is shown in Figure 6C. Only the second and third days are included in the analysis as mice receiving post‐training CNO were untreated up to the end of the first day's training. Learning is compared with the second and third day from the group described in Experiment 2. We found a significant effect of S2‐inhibition (*F*_(1,1)_ = 11.706, *p* < 0.0018) and an interaction between S2‐inhibition and when inhibition was imposed (i.e., before or after training; *F*_(1,1)_ = 5.422, *p* < 0.0266, two‐way ANOVA). Post hoc tests showed that S2 inhibition during training (yellow vs. orange bar) is significantly different from control (*F*_(1,16)_ = 18.27, *p* = 0.0007) while S2 inhibition after training (turquoise vs. blue bar) is not (*F*_(1,17)_ = 0.554, *p* = 0.467). Furthermore, S2 inhibition during training (yellow bar) is the only case not to be different from 50% chance levels of performance (block during training *t*_(9)_ = 0.218, *p* = 0.8321, Cohen's *d* = 0.068; block after training *t*_(7)_ = 2.55, *p* = 0.038, d = 0.90).

The time course of learning within each day was markedly different for the mice receiving S2 inhibition during the training period compared with just after (Figure 6D). While S2‐inhibition during training led to chance levels of performance during each day, mice that only received inhibition after training the previous day improved their performance during the day. We therefore conclude that S2 is required during learning rather than immediately after learning.

### 3.4 Experiment 4

Our results suggested that S2 is required during learning, perhaps because S2 is required for some aspect of execution of what has been learned or, alternatively, because S2 is required for learning itself. To distinguish between these two possibilities, we trained mice to expert levels of performance over a period of 2 days with 40 discrimination trials per day and then tested them on the third day having administered CNO 30 min before the discrimination, so that S2 was inhibited during the task.

**FIGURE 7:**
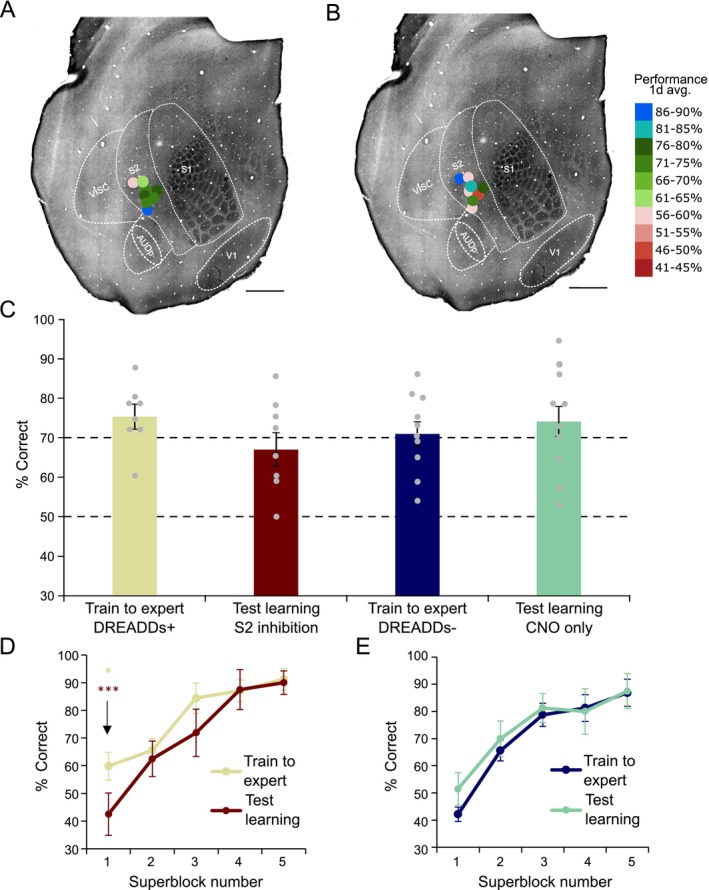
Experiment 4: Effect of S2 inhibition on texture discrimination in expert mice. (A) Injection locations for DREADD. Learning performance averaged over 2 days. (B) Injection locations for the same animals on the single test day with S2 inhibited. Scale bars = 500 μm. (C) After two days of training to expert levels (yellow and blue bars), performance is measured on the 3rd day in mice with DREADD in S2 (red bar) or in animals lacking DREADD but receiving CNO only (green bar). There are no significant effects of S2 inhibition (*F*_(1,1)_ = 0.0699, *p* = 0.7932) nor of sequential days (*F*_(1,1)_ = 0.63, *p* = 0.4327), nor an interaction between the two (*F*_(1,1)_ = 2.65, *p* = 0.1129) by two‐way ANOVA. (D) Time‐course of learning in DREADD mice on the initial training to expertise days (yellow) and on the test day (red). There is an effect of S2 inhibition on first super‐block only (*F*_(1,15)_ = 5.01, *p = 0.0418), which is also significantly different by matched pair *t*‐test (*t*_(7)_ = 3.12, ***p = 0.0083). (E) Time‐course of learning in animals receiving CNO but lacking DREADD injections in S2. There are no significant differences between the training to expertise days (blue) and test day (CNO only green) nor the learning curves for any super‐block, including the first (*F*_(1,19)_ = 2.0076, *p* = 0.1736).

We found that after 2 days of training, mice reached asymptotic levels of discrimination of around 80%–90% correct trials (Figure 7). Performance measured on the third day after prior administration of CNO showed no significant difference between mice with or without DREADD expression in S2 (Figure 7A,B). A two‐way ANOVA showed no significant effects of S2 inhibition (*F*_(1,1)_ = 0.0699, *p* = 0.7932) nor a difference between sequential days (*F*_(1,1)_ = 0.63, *p* = 0.4327), nor an interaction (*F*_(1,1)_ = 2.65, *p* = 0.1129) (Figure 7C).

However, we did notice a small effect on the time course of learning (Figure 7D,E). In expert mice performing the discrimination with S2 inhibited, mice were slower to reach asymptotic levels, as if they were unable initially to recall the task (Figure 7D). This effect was apparent in performance during the first ‘superblock’ of 8 trials, which were significantly different for mice where S2 was inhibited compared with those where it was not (*F*_(1,15)_ = 5.01, *p* = 0.0418, one‐way ANOVA). As the same animals were tested on successive days, we could also look at individual performance and this was also found to be highly significantly different for the first ‘superblock’ between S2 inhibited and control mice (*t*_(7)_ = 3.12, *p* = 0.0083, Cohen's *d* = 3.13, matched pair *t*‐test). Mice quickly learned within the day however and reached similar levels of performance as they had shown on previous days in the absence of S2 inhibition, implying that S2 is not absolutely required for performance of a previously learned task (*t*_(7)_ = 0.133 *p* = 0.45, *d* = 0.133 matched pair *t*‐test). This stands in contrast to the result in naive mice where S2 inhibition prevented learning within the day (Figure 6D) and from day to day (Figure 4). We also found that CNO on its own had no effect on initial recall or execution of the discrimination (Figure 7E). There are no significant differences between the test CNO only (green) and training days (blue) for any ‘superblock’ including the first (*F*_(1,19)_ = 2.0076, *p* = 0.1736). In conclusion, S2 may be involved in initial recall of a previously learned task but does not prevent execution of a previously learned task or improved discrimination performance acquired during the day.

## 4 Discussion

We found that both S1 and S2 are required for texture discrimination learning in mice and that S2 plays a role in the initial stages of learning but is not required once the discrimination has been learned. Once the mice had attained expert levels of discrimination, inhibition of S2 no longer prevented the discrimination nor the improvement in performance during the day (Figure 7). However, even in expert mice, inhibition of S2 did result in lower levels of discrimination for the first few trials in the day, which may reflect impaired recall. Studies in head‐fixed mice using optogenetic inhibition have also suggested that S2 may be involved in recall (Condylis et al. 2020). A plausible explanation for this finding is that once S2 has provided a bridge between the somatosensory and limbic system (Ridley and Ettlinger 1976; Mishkin 1979), sufficient to create expert levels of discrimination, S2 then provides a bridge from the limbic to somatosensory system in recall. We did not find the S2 area to be involved in odour discrimination learning, which implies that its role is specific to touch rather than to some wider role in performing the task.

The S2 area was also not required during the period immediately following training each day, suggesting it may not be required for memory consolidation. In contrast, normal neuronal activity between secondary motor cortex (M2) and S1 during sleep is necessary for memory consolidation (Miyamoto et al. 2016). While it is possible that M2 and S2 feedback connections to S1 play different roles in sleep‐dependent consolidation, it is difficult to form a firm conclusion based on the present results as we did not specifically target neuronal activity during sleep in this study and there may have been an opportunity for normal sleep beyond the 6‐h period of post‐training inhibition that we imposed.

It may not be entirely surprising that S1 is required for tactile learning as the texture discrimination is reliant on the whiskers (Pacchiarini et al. 2020) and the barrel cortex is the major recipient of the somatosensory projection from the whiskers via the brainstem and the thalamus (VPm). Indeed, these findings are consistent with previous studies in rats showing that barrel cortex ablation prevents whisker‐based texture discrimination (Guic‐Robles et al. 1992) and while detection of an object per se does not appear to require S1 (Hong et al. 2018), more complex discriminations do (Ryan et al. 2022). However, S2 also receives a somatosensory thalamic input via POm (Carvell and Simons 1987; Viaene et al. 2011) and a smaller projection from the tails of the VPM barreloids (Pierret et al. 2000), which theoretically could have bypassed the VPm to S1 pathway and enabled learning. Nevertheless, the residual S2 sensory activity was insufficient for learning the texture discrimination when inhibition was increased in S1.

The finding that S2 was also necessary for texture discrimination learning in the presence of normal S1 activity is consistent with the primate literature on S2, which identifies S2 as a vital bridge between S1 and the limbic system, without which tactile learning cannot occur (Ridley and Ettlinger 1976; Mishkin 1979). In primate studies, visual tasks were often used as controls for modality specificity, but here we used odour discrimination, both because it more closely mirrors the tactile discrimination by being performed in the dark and because smell is an ethologically relevant sensory modality for the nocturnal rodent as is touch. Inhibition of S2 had no effect on the ability to learn an odour task showing that S2 plays a role that is specific to tactile learning.

We studied learning in freely moving rather than head‐fixed animals so that they might perform the discrimination more naturally at their own pace and of their own volition. In addition to the benefit of studying more natural behaviour, there is evidence that the animals are less stressed than when head fixed (Juczewski et al. 2020), and because stress hormones such as cortisol can inhibit plasticity (Daw et al. 1991), stress will also presumably affect behavioural evidence of learning too. In favour of this idea, we found animals learned with only 72 trials spaced over 3 days compared with the hundreds of trials typically required for head fixed paradigms over many days (Juczewski et al. 2020). While previous studies have used ablation of cortical areas to study these questions (Guic‐Robles et al. 1992), here we used DREADDs expressed in inhibitory neurons to limit the inhibition to the training periods or the period immediately after training and to lessen the likelihood of compensatory plasticity within the cortical network caused by lesions.

The CNO used to activate the DREADDs does not have off‐target effects at the dose used here (Jendryka et al. 2019; Pandey et al. 2023), and indeed we found that CNO injections in the absence of DREADDs did not prevent learning (Figures 2, 4, 6 and 7). The time course of CNO‐mediated DREADD action was fast enough to be active during the texture discrimination assay; on average it took 20 min to reach a maximum effect and the behavioural testing did not start until 30 min after administration. The effect of DREADD activation was not simply a complete inhibition of excitatory neuronal activity either in S1 or S2, though the effect was sufficient to prevent learning. While sensory responses were decreased during DREADD activation and abolished completely in some neurons, the largest effect of increased inhibition was to alter profoundly network activity.

Both delta‐wave and gamma activity were affected by DREADD‐ mediated inhibition. Delta‐wave activity is known to be highly NMDA receptor dependent (Fox and Armstrong‐James 1986; Armstrong‐James and Fox 1988), and the voltage sensitivity of NMDA receptors (Mayer et al. 1984) would therefore make delta‐wave related network activity particularly vulnerable to increased levels of tonic inhibition produced by DREADD activation in parvalbumin (PV) interneurons. The gamma generation mechanism relies on rhythmic activity in PV interneurons (Buzsaki and Wang 2012), which was converted to tonic firing by the DREADD activation (see Figure 3D), which is why loss of gamma activity was such a sensitive index of the DREADD effect (Figure 3M). It is possible that an inability to produce gamma activity in S1 or S2 contributed to the abolition of tactile learning.

Our results are consistent with the idea that a ‘ventral stream’ might exist for the somatosensory system in transforming primary somatosensory features into representation of an object, such that it can be recalled from memory. In primates, lesions that include S2 are known to produce significant deficits in tactile learning and tactile memory (Ridley and Ettlinger 1976; Garcha and Ettlinger 1979, 1980). However, the lesions produced in these early studies were often large and therefore the results not completely conclusive; for example, ventromedial limbic areas included the temporal pole, amygdala, hippocampus and orbital and prefrontal cortex, and S2 lesions often included parts of the insular cortex. It is also not clear whether areas surrounding the physical ablation were also affected, for example, by inflammation, disruption of blood flow or depolarization block and whether these penumbral areas might have been independently important for the behavioural deficits observed. The spatial accuracy attained in the present study together with the histological verification of the cortical areas affected, using the barrel cortex as a landmark, allows greater confidence in the role of S2 in memory formation.

### 4.1 The Ventral Stream Hypothesis

What are the requirements for establishing whether a hierarchical stream of cortical areas might exist? Ideally one would have anatomical evidence connecting the cortical areas, physiological evidence relating the gradual elaboration of properties from one area to the next and behavioural evidence relating particular links in the stream with the properties in question. Beyond this, one would need to contend with the undoubted existence of both parallel thalamic connectivity and ‘skip’ connections between nonadjacent cortical areas in the stream.

#### 4.1.1 Anatomical Evidence

The essence of the ventral stream pathway can be simply stated as preceding from S1 to S2 to perirhinal cortex (PRh) and hence to entorhinal cortex (ECx) and hippocampus (Hi), and there is good anatomical evidence for the requisite connectivity (Burwell and Amaral 1998). The connections in this putative stream are not unidirectional. Feedback connections also exist between PRh and S2 and PRh and S1 (Agster and Burwell 2009). Back‐projections from PRh and S2 to S1 are likely to be important either for recalling information within the trial itself (working memory) or for recalling information from a previous day's training (reference memory). Detailed tracing studies have shown that lateral ECx in particular projects strongly to the perirhinal cortex (PRh) and a sub‐area PRh (Area 36) then projects strongly to primary (S1) and secondary somatosensory (S2) cortex (Witter et al. 1989; Agster and Burwell 2009). Both S2 and PRh cortex project to layer 1 of S1 (Burwell and Amaral 1998; Schuman et al. 2021). Inhibition of either S1 or S2 as presented in this study therefore temporarily breaks this information stream and prevents texture learning either by preventing a representation of the object from forming or from preventing it from being recalled.

#### 4.1.2 Physiological Evidence

Both primary (S1) and secondary (S2) somatosensory cortex contain neurons that carry information about texture (Jadhav and Feldman 2010; Safaai et al. 2013; Garion et al. 2014; Ramos 2014; Zuo et al. 2015; Lieber and Bensmaia 2019). However, while many neurons carry information about texture in S1, few appear specifically to code for it (Garion et al. 2014; Buetfering et al. 2022). In S2, most neurons respond in a manner that combines sensory and categorical information, for example about different vibration sequences (Rossi‐Pool et al. 2021). This leads to the idea that S2 may elaborate information received from S1 to build a percept of an object. The idea invites comparison with the ventral stream in the visual system where elementary visual features are combined and filtered through a hierarchy of cortical areas to produce more complex representations, eventually of objects through corticocortical processing (Mishkin and Ungerleider 1982). In particular, S2 neurons have been found to represent the difference between two independently presented tactile features when the reward depends on that difference (Romo et al. 2002) and to hold a trace of the first feature of the discriminated pair during encounter with the second (Salinas et al. 2000). Finally, there is evidence to suggest that S2 neurons recall information in anticipation of touch (Condylis et al. 2020). The latter property of working memory and recall is highly likely to be important in the behavioural discrimination we have studied, where mice learn to reduce the errors they make by digging in the unbaited bowl by adopting a strategy of making several visits to each bowl before choosing.

#### 4.1.3 Behavioural Evidence

In this study, we provide evidence that the first two cortical areas in the putative stream are relevant to learning a texture discrimination. The functional ablations were bilateral to avoid any interhemispheric transfer given that S2 receptive fields are bilateral. Our studies are consistent with cortical ablation studies in S1 (Guic‐Robles et al. 1992) that prevented texture discrimination and with studies involving optogenetic inhibition of S2 that have shown S2 to be necessary for discriminating between different sequences of tactile stimuli (Bale et al. 2021).

#### 4.1.4 Parallel Versus Serial Processing

Theoretically, individual inhibition of either S1 or S2 might have been compensated by activity in the other if the parallel processing pathways from the thalamus carried sufficient information to either cortical area. Conversely, a serial processing stream might be indicated if inhibition of either area prevented texture discrimination. Our studies provide quite strong evidence that parallel pathways from the thalamus to either S1 (Lu and Lin 1993), S2 (Carvell and Simons 1987; Pierret et al. 2000) or PRh (Burwell and Amaral 1998) that might short‐circuit the serial pathway are insufficient to compensate for the serial connections between S1 and S2 and areas downstream leading to the hippocampus, at least for this texture task. It is not clear why S2 cannot facilitate tactile learning on its own given its thalamic input. However, POm does receive a large component of its output from S1, which once inhibited would degrade the information it sends to S2 (Hoogland et al. 1987; Diamond et al. 1992). Furthermore, S2 receptive fields tend to have a lower frequency response which may not represent texture as well as S1 (Kwegyir‐Afful and Keller 2004). Thus it is conceivable that a different simpler discrimination might survive S1 inhibition.

Given that ‘skip’ connections also exist between PRh and S1 that were not directly affected by our DREADD inhibition of S2, we conclude that these connections are also insufficient for learning in and of themselves and some feature of S2 processing, either between S2 and S1 or between S2 and downstream structures are necessary for texture discrimination learning. Recent studies have shown that PRh cortical projections to S1 are correlated with burst behaviour in layer 5 neurons that is in turn correlated with learning the occurrence of an electrical stimulus delivered directly to the barrel cortex (Doron et al. 2020). While this pathway may be important for learning, from the evidence presented here, it does not appear to be able to compensate for the role played by S2.

## 5 Conclusion

Our studies provide evidence that S1 and S2 are both necessary for tactile discrimination learning in mice. The findings are not consistent with the notion that parallel pathways from thalamus to cortex can compensate for the lack of corticocortical interaction. Rather, the findings are consistent with the theory of a ventral stream linking somatosensory cortex to the limbic system to enable elaboration of sensory processing, learning and association of a reward with a tactile feature.

## Author Contributions

**Anurag Pandey:** data curation, formal analysis, investigation, methodology, writing – review and editing. **Sungmin Kang:** data curation, formal analysis, investigation, methodology, software, writing – review and editing. **Nicole Pacchiarini:** data curation, formal analysis, investigation, methodology, visualization, writing – review and editing. **Hanna Wyszynska:** data curation, investigation, visualization, writing – review and editing. **Zena Masseri:** investigation, methodology, visualization. **Joseph O'Neill:** formal analysis, funding acquisition, investigation, methodology, project administration, software, supervision, validation, visualization, writing – review and editing. **R. Honey:** conceptualization, formal analysis, funding acquisition, supervision, validation, writing – original draft, writing – review and editing. **Kevin Fox:** conceptualization, data curation, formal analysis, funding acquisition, investigation, methodology, project administration, supervision, validation, writing – original draft, writing – review and editing.

## Funding

This research was supported by the Medical Research Council (MR/W004844/1; KF), Biotechnology and Biological Sciences Research Council (BB/T007028/1; KF, RCH, JON) and Biotechnology and Biological Sciences Research Council SWBio PhD studentships (NP, HW).

## Ethics Statement

The experiments reported in this study copy with the UK Animal (Scientific Procedures) Act 1986.

## Conflicts of Interest

The authors declare no conflicts of interest.

## Supporting information


**Figure S1:** Effect of DREADDs on delta wave activity in S1. Delta power (0.5‐4 Hz) was calculated continuously from 60 min before to 60 min after CNO application in overlapping (50%) 13.1 s windows. The DREADD construct was located in layer 4 of either S1 (A‐C) or S2 (D‐F). **A:** Delta power in a single S1 layer 4 channel in animal 1. Vertical line: time of CNO injection, grey box: baseline (pre CNO) epoch. Note: delta power rapidly drops after CNO injection. **B:** Power was normalised using the mean and standard deviation of delta activity during base line recording (grey box), to provide an instantaneous change in power from baseline (z score). These signals were averaged across electrodes located in layer 4 of S1 of animal 1. Red shaded region: ±SEM **C:** Mean change in delta power (mean post‐CNO *z* score) after CNO injection in 8 S1 channels and 8 S2 channels are shown (filled circles) for each animal, as well as their means. Error bars ±SEM. **D‐E:** DREADD activation in S2 reduces delta power in layer 4 S2 electrodes (calculated as shown in **A,B**). Note: activation of DREADD in S2 is followed by a reduction in delta power in S2 electrodes. **F:** Mean change in power delta power following DREADD activation in S2 are shown for 8 electrodes in S1 and S2, for each animal.

**Figure S2:** Anatomical location of the electrode track relative to cortical layers and cortical areas. **A:** Coronal section of PV‐Cre mouse brain showing DREADD expression of td‐tomato in S1 barrel field (red) and electrode track labelled with DiI (green, painted on the electrode prior to insertion). Note that some neurones are labelled in layer 5 of S2 by the DiI (green). **B:** The cortical layers were estimated by superimposing the nearest section in the Allen mouse brain Atlas on the histological section and then making small adjustments to allow for oedema where present using local cytoarchitectural features distinguishing the granular layer from pyramidal cell extragranular layers. Abbreviations: **S1tr** primary somatosensory cortex trunk representation, **S1bf** primary somatosensory cortex barrel field, **S2** second somatosensory cortex, **VISC** visceral area, **AIP** anterior insular area, **cc** white matter including corpus callosum, **VL** lateral ventricle, **int** internal capsule, **GP** globes palidus, **CP** caudate putamen, **TH** thalamus. All other abbreviations in **B** as given in the Allen brain cell atlas from which it is adapted. Adapted from the Allen Reference Atlas—Mouse Brain at the slice position 66 (AP ‐0.95). Allen Mouse Brain Atlas, mouse.brain‐map.org and atlas.brain‐map.org.

**Figure S3:** Change in firing rate distributions for spontaneous activity and evoked activity produced by DREADD‐induced increased inhibition. **Top:** Five cases of spontaneous activity changes are shown, three where DREADD is active in S1 and two in S2. Note the increase in the proportion of low firing rate cells after CNO injection (red line) compared with control (black line). The effect is even greater in S2 than S1. **Bottom:** The same five cases are shown again but for whisker deflection evoked responses. D value indicates the Kolmogorov–Smirnov value of greatest divergence.

**Figure S4:** Effect of DREADDs on sensory responses in S1. **A‐E** (1–7): Examples of the variety of effects of DREADD (red trace) on control firing rate (black). Examples are roughly grouped by inter‐interval autocorrelogram (left of each pair). **A1‐A7** and **B1–2** show bursting cells where 6/9 are strongly inhibited. **B2–7** neurones with longer intra‐burst intervals 4/5 of which are strongly inhibited. Some cells showed high spontaneous activity which was inhibited by DREADD activation (D1–4) three of which had an inhibitory response to stimulation. Two cells are shown that increased their firing rate **E1,2** with DREADD activation and are presumably PV neurones. One cell shows an inhibitory response (**E1**) and the other retained its excitatory response to stimulation at a lower signal to noise ratio **E2**.

**Data S1:** Supporting Information.

## Data Availability

The behavioural data that support the findings of this study are available in a depository. The electrophysiological data is available on reasonable request.
