# Supplementary material for: Secondary Somatosensory Cortex Is Required for Learning but Not Execution of a Tactile Discrimination
Source: Eur J Neurosci. 2026 Jan 29;63(3):e70390. doi: 10.1111/ejn.70390 (PMC12853412; doi:10.1111/ejn.70390)
Supplement: Supplementary file 1 — Figure S1: Effect of DREADDs on delta wave activity in S1. Delta power (0.5‐4 Hz) was calculated continuously from 60 min before to 60 min after CNO application in overlapping (50%) 13.1 s windows. The DREADD construct was located in layer 4 of either S1 (A‐C) or S2 (D‐F). A: Delta power in a single S1 layer 4 channel in animal 1. Vertical line: time of CNO injection, grey box: baseline (pre CNO) epoch. Note: delta power rapidly drops after CNO injection. B: Power was normalised using the mean and standard deviation of delta activity during base line recording (grey box), to provide an instantaneous change in power from baseline (z score). These signals were averaged across electrodes located in layer 4 of S1 of animal 1. Red shaded region: ±SEM C: Mean change in delta power (mean post‐CNO z score) after CNO injection in 8 S1 channels and 8 S2 channels are shown (filled circles) for each animal, as well as their means. Error bars ±SEM. D‐E: DREADD activation in S2 reduces delta power in layer 4 S2 electrodes (calculated as shown in A,B). Note: activation of DREADD in S2 is followed by a reduction in delta power in S2 electrodes. F: Mean change in power delta power following DREADD activation in S2 are shown for 8 electrodes in S1 and S2, for each animal. [file EJN-63-0-s001.pdf]

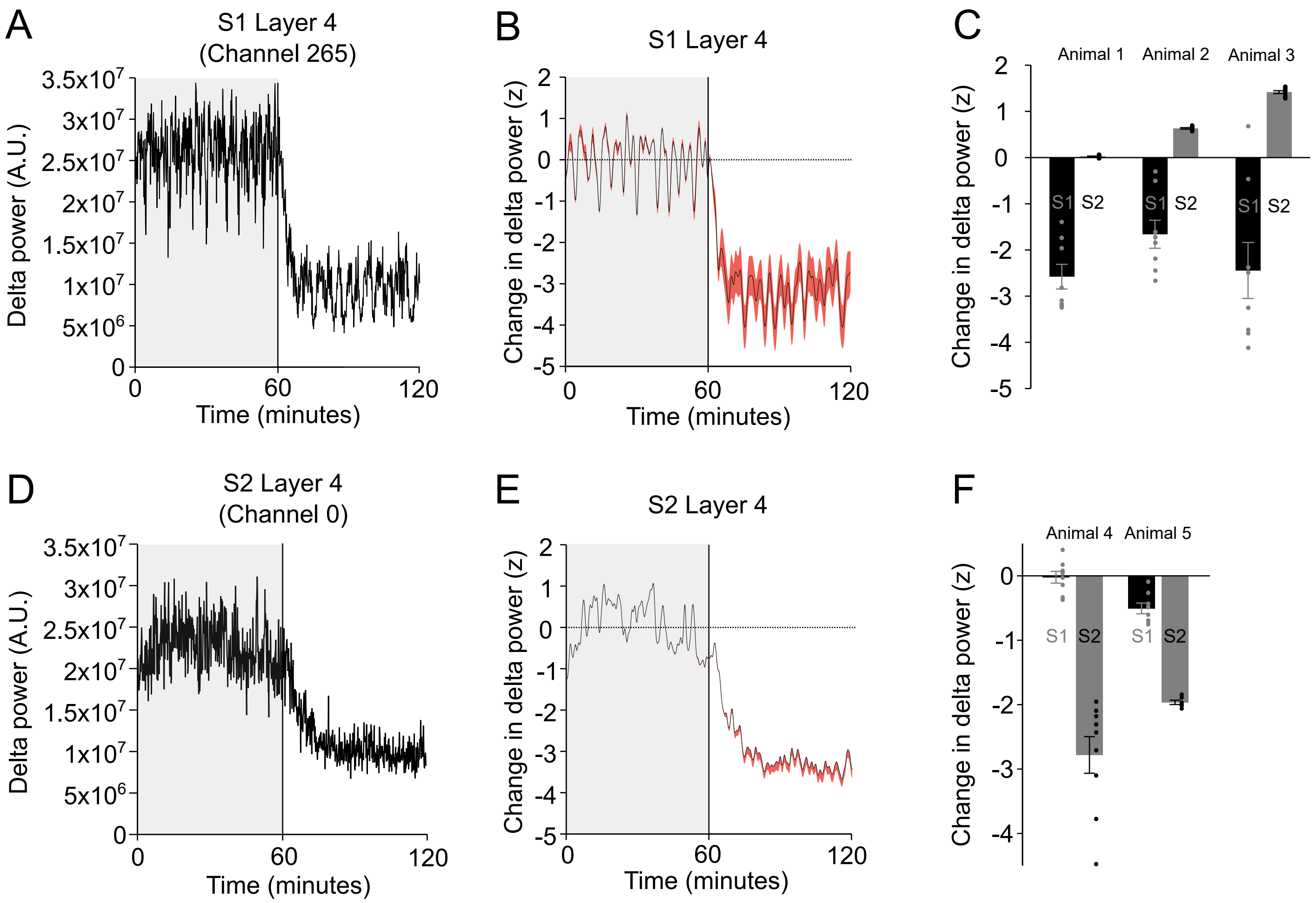

Figure S1. DREADD activation profoundly reduces delta power. Delta power (0.5-4Hz) was calculated continuously from 60 minutes before to 60 minutes after CNO application in overlapping (50%) 13.1s windows. The DREADD construct was located in layer 4 of either S1 (A-C) or S2 (D-F). **A:** Delta power in a single S1 layer 4 channel in animal 1. Vertical line: time of CNO injection, grey box: baseline (pre CNO) epoch. Note: delta power rapidly drops after CNO injection. **B:** Power was normalised using the mean and standard deviation of delta activity during base line recording (grey box), to provide an instantaneous change in power from baseline (z score). These signals were averaged across electrodes located in layer 4 of S1 of animal 1. Red shaded region: ±SEM. **C:** Mean change in delta power (mean post-CNO z-score) after CNO injection in 8 S1 channels and 8 S2 channels are shown (filled circles) for each animal, as well as their means. Error bars ±SEM. **D-E:** DREADD activation in S2 reduces delta power in layer 4 S2 electrodes (calculated as shown in A & B). Note: activation of DREADD in S2 is followed by a reduction in delta power in S2 electrodes. **F:** Mean change in power delta power following DREADD activation in S2 are shown for 8 electrodes in S1 and S2, for each animal.
