# Supplementary material for: Secondary Somatosensory Cortex Is Required for Learning but Not Execution of a Tactile Discrimination
Source: Eur J Neurosci. 2026 Jan 29;63(3):e70390. doi: 10.1111/ejn.70390 (PMC12853412; doi:10.1111/ejn.70390)
Supplement: Supplementary file 2 — Figure S2: Anatomical location of the electrode track relative to cortical layers and cortical areas. A: Coronal section of PV‐Cre mouse brain showing DREADD expression of td‐tomato in S1 barrel field (red) and electrode track labelled with DiI (green, painted on the electrode prior to insertion). Note that some neurones are labelled in layer 5 of S2 by the DiI (green). B: The cortical layers were estimated by superimposing the nearest section in the Allen mouse brain Atlas on the histological section and then making small adjustments to allow for oedema where present using local cytoarchitectural features distinguishing the granular layer from pyramidal cell extragranular layers. Abbreviations: S1tr primary somatosensory cortex trunk representation, S1bf primary somatosensory cortex barrel field, S2 second somatosensory cortex, VISC visceral area, AIP anterior insular area, cc white matter including corpus callosum, VL lateral ventricle, int internal capsule, GP globes palidus, CP caudate putamen, TH thalamus. All other abbreviations in B as given in the Allen brain cell atlas from which it is adapted. Adapted from the Allen Reference Atlas—Mouse Brain at the slice position 66 (AP ‐0.95). Allen Mouse Brain Atlas, mouse.brain‐map.org and atlas.brain‐map.org. [file EJN-63-0-s004.pdf]

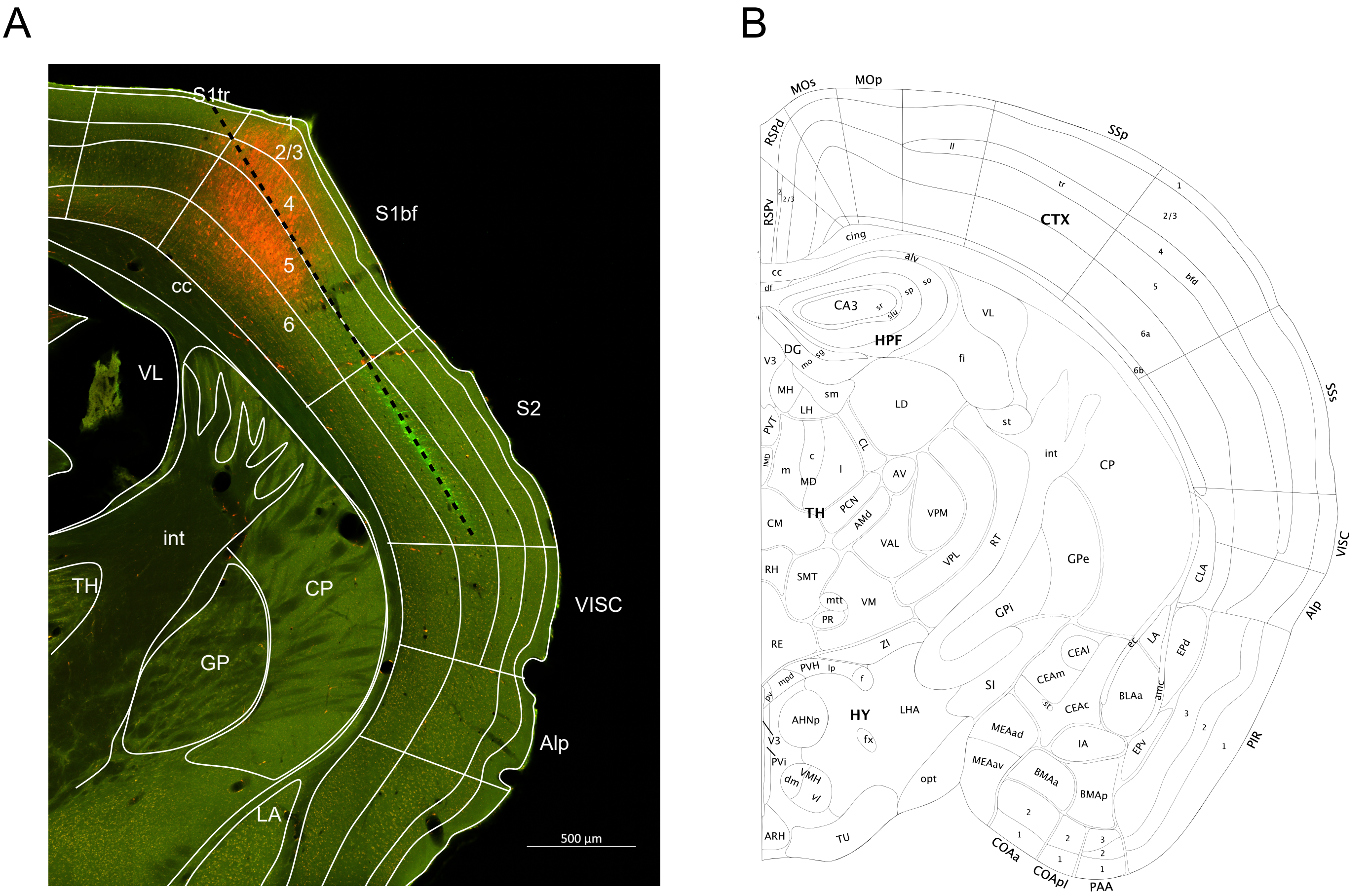

Figure S2. Anatomical location of the electrode track relative to cortical layers and cortical areas.
A: Coronal section of PV-Cre mouse brain showing DREADD expression of td-tomato in S1 barrel field (red) and electrode track labelled with DiI (green, painted on the electrode prior to insertion). Note that some neurones are labelled in layer 5 of S2 by the DiI (green). **B:** The cortical layers were estimated by superimposing the nearest section in the Allen mouse brain Atlas on the histological section and then making small adjustments to allow for oedema where present using local cytoarchitectural features distinguishing the granular layer from pyramidal cell extragranular layers. Adapted from the Allen Reference Atlas – Mouse Brain at the slice position 66 (AP -0.95). Allen Mouse Brain Atlas, mouse.brain-map.org and atlas.brain-map.org. Abbreviations: **S1tr** primary somatosensory cortex trunk representation, **S1bf** primary somatosensory cortex barrel field, **S2** second somatosensory cortex, **VISC** visceral area, **AIP** anterior insular area, **cc** white matter including corpus callosum, **VL** lateral ventricle, **int** internal capsule, **GP** globes palidus, **CP** caudate putamen, **TH** thalamus. All other abbreviations in B as given in the Allen brain cell atlas from which it is adapted.
