# Supplementary material for: Secondary Somatosensory Cortex Is Required for Learning but Not Execution of a Tactile Discrimination
Source: Eur J Neurosci. 2026 Jan 29;63(3):e70390. doi: 10.1111/ejn.70390 (PMC12853412; doi:10.1111/ejn.70390)
Supplement: Supplementary file 3 — Figure S3: Change in firing rate distributions for spontaneous activity and evoked activity produced by DREADD‐induced increased inhibition. Top: Five cases of spontaneous activity changes are shown, three where DREADD is active in S1 and two in S2. Note the increase in the proportion of low firing rate cells after CNO injection (red line) compared with control (black line). The effect is even greater in S2 than S1. Bottom: The same five cases are shown again but for whisker deflection evoked responses. D value indicates the Kolmogorov–Smirnov value of greatest divergence. [file EJN-63-0-s002.pdf]

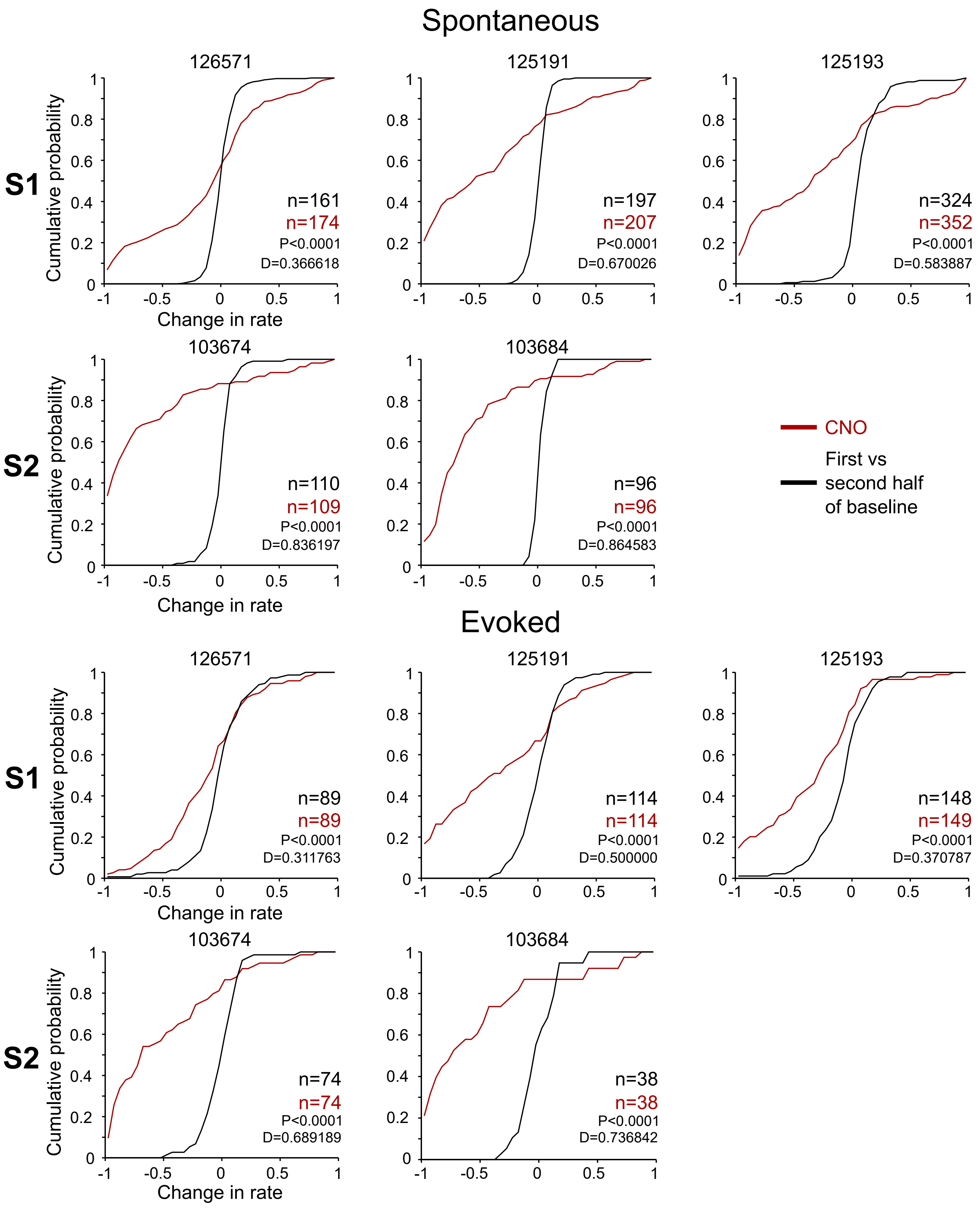

Figure S3 Change in firing rate distributions for spontaneous activity and evoked activity produced by DREADD-induced increased inhibition. **Top:** Five cases of spontaneous activity changes are shown, three where DREADD is activity in S1 and two in S2. Note the increase in the proportion of low firing rate cells after CNO injection (red line) compared with control (black line). The effect is even greater in S2 than S1. **Bottom:** The same five cases are shown again but for whisker deflection evoked responses. D value indicates the Kolmogorov-Smirnov value of greatest divergence.
