# Supplementary material for: Secondary Somatosensory Cortex Is Required for Learning but Not Execution of a Tactile Discrimination
Source: Eur J Neurosci. 2026 Jan 29;63(3):e70390. doi: 10.1111/ejn.70390 (PMC12853412; doi:10.1111/ejn.70390)
Supplement: Supplementary file 4 — Figure S4: Effect of DREADDs on sensory responses in S1. A‐E (1–7): Examples of the variety of effects of DREADD (red trace) on control firing rate (black). Examples are roughly grouped by inter‐interval autocorrelogram (left of each pair). A1‐A7 and B1–2 show bursting cells where 6/9 are strongly inhibited. B2–7 neurones with longer intra‐burst intervals 4/5 of which are strongly inhibited. Some cells showed high spontaneous activity which was inhibited by DREADD activation (D1–4) three of which had an inhibitory response to stimulation. Two cells are shown that increased their firing rate E1,2 with DREADD activation and are presumably PV neurones. One cell shows an inhibitory response (E1) and the other retained its excitatory response to stimulation at a lower signal to noise ratio E2. [file EJN-63-0-s003.pdf]

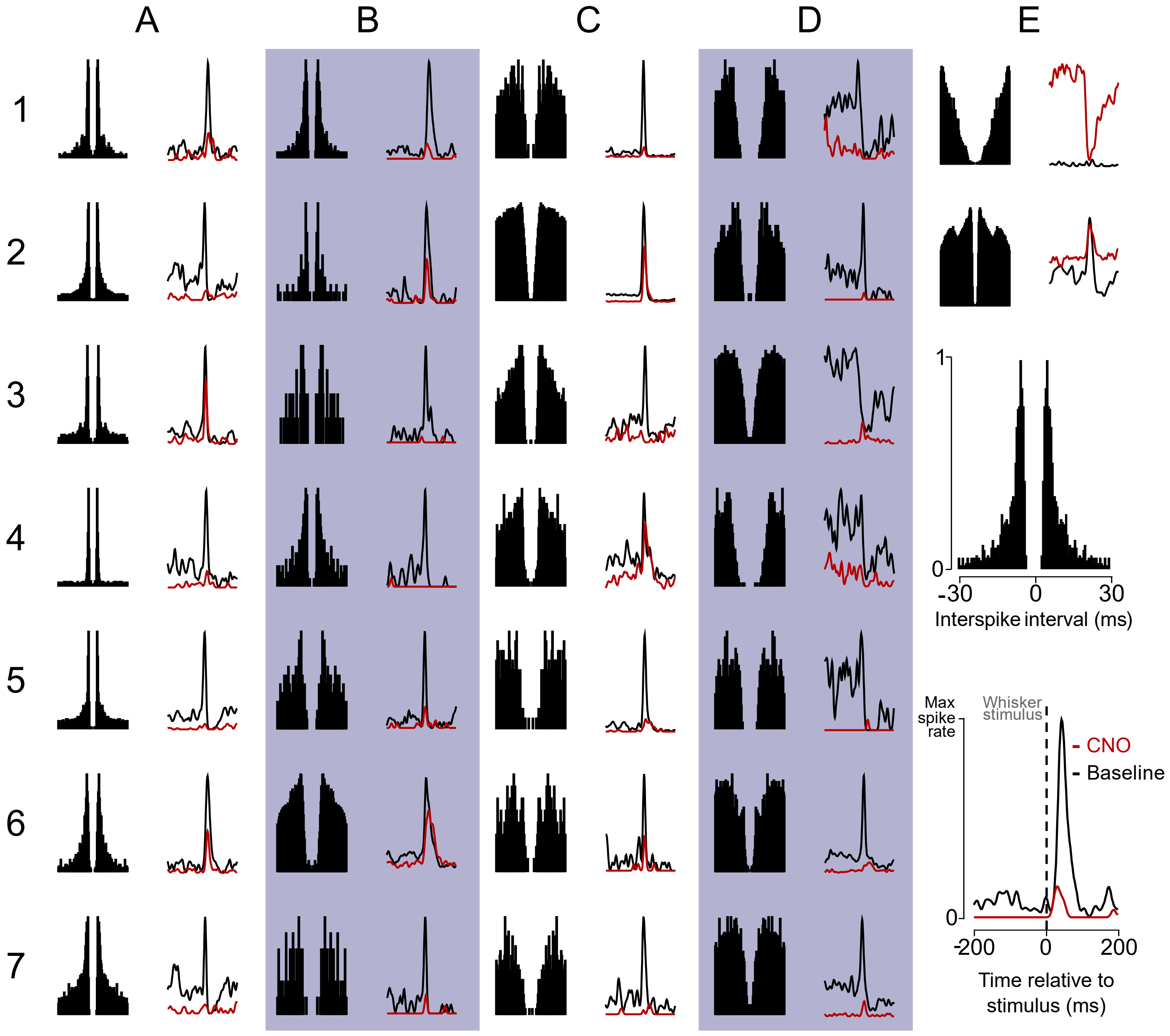

Figure S4. Effect of DREADDs on sensory responses in S1. **A-E (1-7):** Examples of the variety of effect of DREADD (red trace) on control firing rate (black). Examples are roughly grouped by inter-interval autocorrelogram (left of each pair). A1-A7 and B1-2 show bursting cells where 6/7 are strongly inhibited. B 2-7 neurones with longer intra-burst intervals 4/5 of which are strongly inhibited. Some cells showed high spontaneous activity which was inhibited by DREADD activation (D1-4) three of which had an inhibitory response to stimulation. Two cells are shown that increased their firing rate E1,2 with DREADD activation and are presumably PV neurones. One cell shows an inhibitory response (E1) and the other retained its excitatory response to stimulation at a lower signal to noise ratio E2.
